# Tuning Size and Morphology of mPEG-*b*-p(HPMA-Bz) Copolymer Self-Assemblies Using Microfluidics

**DOI:** 10.3390/polym12112572

**Published:** 2020-11-02

**Authors:** Jaleesa Bresseleers, Mahsa Bagheri, Coralie Lebleu, Sébastien Lecommandoux, Olivier Sandre, Imke A. B. Pijpers, Alexander F. Mason, Silvie Meeuwissen, Cornelus F. van Nostrum, Wim E. Hennink, Jan C.M. van Hest

**Affiliations:** 1 Department of Bio-Organic Chemistry, Eindhoven University of Technology, 5600 MB Eindhoven, The Netherlands; j.bresseleers@tue.nl (J.B.); i.a.b.pijpers@tue.nl (I.A.B.P.); a.f.mason@tue.nl (A.F.M.); 2 Ardena Oss, 5349 AB Oss, The Netherlands; silvie.meeuwissen@ardena.com; 3 Department of Pharmaceutics, Utrecht Institute for Pharmaceutical Sciences (UIPS), Faculty of Science, Utrecht University, 3508 TB Utrecht, The Netherlands; m.bagheri@uu.nl (M.B.); c.f.vannostrum@uu.nl (C.F.v.N.); w.e.hennink@uu.nl (W.E.H.); 4 Laboratoire de Chimie de Polymères Organiques, Université de Bordeaux, UMR 5629 CNRS, Bordeaux-INP, 33600 Pessac, France; Coralie.Lebleu@gmail.com (C.L.); Lecommandoux@enscbp.fr (S.L.); olivier.sandre@enscbp.fr (O.S.)

**Keywords:** block copolymers, nanoparticles, micelles, polymersomes, HPMA, size control, nanoprecipitation, microfluidics, micromixer

## Abstract

The careful design of nanoparticles, in terms of size and morphology, is of great importance to developing effective drug delivery systems. The ability to precisely tailor nanoparticles in size and morphology during polymer self-assembly was therefore investigated. Four poly(ethylene glycol)-*b*-poly(*N*-2-benzoyloxypropyl methacrylamide) mPEG-*b*-p(HPMA-Bz) block copolymers with a fixed hydrophilic block of mPEG 5 kDa and a varying molecular weight of the hydrophobic p(HPMA-Bz) block (A: 17.1, B: 10.0, C: 5.2 and D: 2.7 kDa) were self-assembled into nanoparticles by nanoprecipitation under well-defined flow conditions, using microfluidics, at different concentrations. The nanoparticles from polymer A, increased in size from 55 to 90 nm using lower polymer concentrations and slower flow rates and even polymer vesicles were formed along with micelles. Similarly, nanoparticles from polymer D increased in size from 35 to 70 nm at slower flow rates and also formed vesicles along with micelles, regardless of the used concentration. Differently, polymers B and C mainly self-assembled into micelles at the different applied flow rates with negligible size difference. In conclusion, this study demonstrates that the self-assembly of mPEG-*b*-p(HPMA-Bz) block copolymers can be easily tailored in size and morphology using microfluidics and is therefore an attractive option for further scaled-up production activities.

## 1. Introduction

During the past few decades, polymeric-based drug delivery nanoparticles, in particular polymeric micelles, have received growing interest for tumor targeting and other therapeutic purposes [1,2,3]. In general, polymeric micelles are core-shell structures composed of amphiphilic block copolymers. The shell consists of a brush of the hydrophilic block chains, usually poly(ethylene glycol) (PEG), which provides stealth-like properties against non-specific protein adsorption and offers good colloidal stability in physiological conditions. The hydrophobic core, in turn, can be used to accommodate poorly water-soluble drugs [4,5,6]. A careful design of the topological features of the polymeric micelles is of importance to achieve efficacy of treatment e.g., regarding pharmacokinetics and tumor penetration [7,8,9].

The morphological characteristics of nanoparticles such as size and shape have a determinant effect on their in vivo and in vitro performance [10]. In general, the morphology of nanoparticles can impact drug loading and release, pharmacokinetics and biodistribution, cell uptake and biosafety features [11,12]. It was shown in preclinical studies that nanoparticles with sizes < 100 nm could extravasate better to target tumors and those with sizes < 50 nm could even penetrate the tumor deeper, exerting better tumor efficacy [7,9,13]. In terms of shape, many biological processes such as pharmacokinetics and cell uptake could be influenced [10,14]. For instance, it was shown that cellular uptake was more inhibited by nanoparticles with higher aspect ratios compared to spherical particles. So far, polymersomes and micelles are the most frequently studied and advanced polymer-based nanomedicines for cancer therapy. However, achieving a full control over the self-assembly of block copolymer chains into particles still remains a challenge [11].

Various methods are available to prepare polymeric micelles such as emulsion-based and solvent displacement procedures. The latter, first introduced in the late nineties by Devissaguet and Fessi [15], is also referred to as nanoprecipitation, which renders tailorable characteristics such as size and size distribution [16]. The nanoprecipitation method is a simple, fast and straightforward technique to produce polymer-based nanoparticles. In short, an amphiphilic block copolymer (possibly in combination with a drug) is dissolved in a water-miscible organic solvent. The obtained solution is then added to an aqueous phase, which acts as a non-solvent for the hydrophobic block and leads to the formation of (drug-loaded) nanoparticles. In the final step, the organic solvent is removed by evaporation or dialysis [17,18,19,20].

The conventional nanoprecipitation method is performed in batch mode i.e., in traditional glassware, which is simple and efficient. Nevertheless, it has its limitations regarding uniformity and reproducibility of mixing. For instance, temperature or concentration inhomogeneity during mixing can have a substantial effect on the final size and structure of the particles [16]. Such issues might be particularly relevant with a block copolymer such as poly(ethylene glycol)-*b*-poly(*N*-2-benzoyloxypropyl methacrylamide) (mPEG-*b*-p(HPMA-Bz)), since the benzyl groups have shown to provide strong ᴨ-ᴨ stacking interactions and its self-assembly most likely leads to kinetically trapped nanoparticles rather than a dynamic micelle state [21]. Even for a block copolymer without aromatic groups such as poly(ethylene glycol)-*block*-poly(butyl acrylate) (mPEG-*b*-PBMA), previously reported simulations demonstrated that its self-assembly is controlled by kinetics and the applied process conditions rather than thermodynamics [22]. In this case, with a moderately hydrophobic block, the introduction of charges in the hydrophilic block can drive the self-assembly towards dynamic micelles [23].

Microfluidics is a technology that handles minute volumes of solutions in microscale fluidic devices in a precise and controlled way [24,25], usually in a laminar flow regime. As a comparison, flash nanoprecipitation in high pressure reactors rather uses turbulent flows that enable them to reach the shortest mixing times of the solvent and non-solvent [16,19], which is a way to separate nucleation from particle growth [26], and ultimately better controlling the size distribution of the self-assemblies. It is usually hypothesized that small dimensions of channels lead to a much higher surface to volume ratio of the solutions to be mixed than what is achieved in macroscopic vessels, which in turn reduces the diffusional times. Thus, controlled and tunable mixing is expected to give access to a kinetically controlled nanoprecipitation process, facilitating control over size and size distribution of the formed self-assemblies [27,28]. Therefore, microfluidics has been evaluated in the literature to see if it can be considered as a reliable and up-scalable technology to control the self-assembly of polymeric nanoparticles [29,30,31]. A self-assembly process is highly dependent upon both external (e.g., temperature) and internal parameters such as interfacial and viscous forces. Parameters such as fluid viscosity and mass density are homogeneous at the scale of microchannel dimensions, therefore one expects that the self-assembly process can be efficiently controlled by flow rates of, respectively, the solvent and anti-solvent [29]. Previous works reported only moderate reduction of size dispersities, but showed at least real optimization in terms of drug encapsulation rates [32]. For instance, Xu et al. described a lab-made coaxial flow chip enabling encapsulation of hydrophobic drugs with high efficiency in poly(lactic-*co*-glycolic acid) (PLGA) nanoparticles [33]. Furthermore, it is important to remark that microfluidics has a great potential in scaling up production of polymer-based nanomedicines thanks to its continuous flow operational process, which is a major advantage for the production of formulations when moving to clinical (trial) applications [34,35,36].

Although there is still some variability in outcome, most of the previously published studies showed that, when using microfluidics, fine-tuning of the flow rates and the ratio of organic solvent to the aqueous buffer enables control over both final particle size and polydispersity index (PDI) [36,37,38,39,40,41]. As an example, for the preparation of chitosan nanoparticles using microfluidics, varying the flow rates of the polymeric to alkaline water solutions resulted in the formation of smaller nanoparticles of 63 and 102 nm at, respectively, the shortest and longest applied mixing time in the microfluidic device, as compared to 161 nm nanosized particles using bulk production [37]. In the same study, it was also observed that the nanoparticles obtained from microfluidics had a narrower size distribution over all applied mixing times compared to the particles prepared using a bulk procedure. Similarly, Bally et al. reported that increasing the flow rates of non-solvent to the polymer solution and thus a faster and more efficient mixing resulted in smaller poly(methyl methacrylate)-based nanoparticles compared to particles prepared in a batch process at similar solvent to non-solvent ratios (100 and 245 nm, respectively) [38]. In general, microfluidic devices offer control over flow rates, and therefore mixing times, which is of utmost importance to control the self-assembly and to tailor particle size [29,36,39,40,41].

Previously, we reported on the preparation of size-tunable micelles based on poly(ethylene glycol)-*block*-poly(N-2-benzoyloxypropyl methacrylamide) (mPEG-*b*-p(HPMA-Bz)) in batch mode [42]. The study showed that the obtained micelles exhibited crew-cut structures and that their sizes were sensitive to the mixing rate of solvents and non-solvents, emphasizing the need for a system with robust mixing features. Therefore, in the present study, a microfluidic mixing device was used to investigate the effects of process and formulation parameters on the size of mPEG-*b*-p(HPMA-Bz) micelles. It was shown that the self-assembly of mPEG-*b*-p(HPMA-Bz) block copolymers could be easily tailored in size and morphology. This is of great importance with our aim of achieving a robust method for the production of small (<100 nm) and well-defined polymeric nanoparticles eventually suitable for drug delivery purposes. More precisely, a commercial glass chip from Dolomite Inc. was used, which belongs to the herringbone-type micromixers employing chaotic laminar flow [43]. This set-up had previously shown its suitability for achieving morphological control via the assembly of block copolymers with respectively poly(trimethylene carbonate) and poly(*ɣ*-benzyl-*L*-glutamate) as the hydrophobic block, and respectively poly(ethylene oxide) and elastin-like polypeptide (ELP) as the hydrophilic block, on the very same chip [41,44].

## 2. Materials and Methods

### 2.1. Materials

*N*-(2-benzoyloxypropyl) methacrylamide (HPMA-Bz) monomer and methoxy-poly(ethylene glycol)-(4,4-azobis(4-cyanopentanoic acid)-methoxy-poly(ethylene glycol) (mPEG-ABCPA-mPEG) macroinitiator (each mPEG block with a molecular weight of 5.0 kDa) were synthesized and characterized using previously published protocols [42,45,46]. Poly(tetrafluoroethylene) (PTFE) and cellulose acetate syringe disc filters (both 0.22 µm) and bovine serum albumin (BSA) were obtained from Merck (Darmstadt, Germany). PEG standards for gel permeation chromatography (GPC) analysis were obtained from Agilent (Santa Clara, CA, USA). All solvents were purchased from commercial suppliers and used as received.

### 2.2. Instrumentation

#### Laminar Chaotic Mixing Microfluidic System

**Figure 1:**
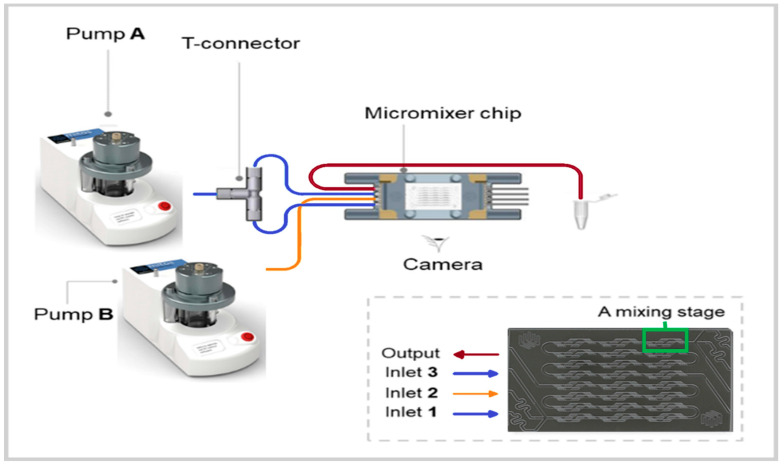
Scheme of the microfluidic system used in the present study (from Dolomite Inc., Royston, UK) [47].

The core of the microfluidic system consists of a commercial herringbone micromixer glass chip (Part No. 3200401 purchased from Dolomite Center Ltd., Royston, UK). According to the manufacturer, the chip consists of two independent channels with 12 mixing steps with a depth and width alternating between 125 × 350 and 50 × 125 µm^2^, creating lamination of the entering flows and even swirling of the flow streams. The whole microfluidic system is constituted of two pressure pumps and two flowmeters (range 30–1000 µL/min) connected to a computer to control the pumps with the provided software (Mitos Flow Control Center 2.5.17 software), PTFE tubing, an ethylene tetrafluoroethylene (ETFE) T-connector, a micromixer chip and a fast camera from Dolomite Microfluidics^®^ (Figure 1). Pump A was linked to the chip through inlets 1 + 3 using the T-connector, whereas the pump B was connected directly to inlet 2. Flow rate calibration as a function of applied pressure and mixing time calculation was done as described in the manual provided by the supplier (Supplementary Information Figure S5 and Table S1).

### 2.3. Methods

#### 2.3.1. Dynamic Light Scattering (DLS) Analysis

The hydrodynamic diameter of the self-assemblies was determined by DLS analysis using a Malvern Zetasizer nano series ZS90 (Malvern, UK) with a measurement angle of 173° and a temperature of 25 °C. Prior to measuring, the samples were filtered using a 0.22 µm cellulose acetate disk filter to remove any dust and large particles.

#### 2.3.2. Asymmetric Flow Field-Flow Fractionation Connected to Multi-Angle Laser Light Scattering Detector (AF4-MALLS)

The radius of gyration (R_g_) was determined using a Wyatt Dualtec AF4 instrument connected to a Shimadzu LC-2030 Prominence-I system with a Shimadzu LC-2030 auto-sampler. The fractionation was accomplished on an AF4 short channel with a spacer of 350 µm and a 10 kDa membrane of regenerated cellulose. The AF4 was attached to a light scattering detector (Wyatt DAWN HELEOS II, Santa Barbara, CA, USA) that was installed at 16 different angles ranging from 12.9° to 157.8° using a laser operating at 664.5 nm and a refractive index detector (Wyatt Optilab, Santa Barbara, CA, USA). BSA (5 mg/mL) dissolved in phosphate buffer saline (PBS) (0.01 M phosphate buffer, 0.0027 M potassium chloride and 0.137 M sodium chloride, pH 7.4, at 25 °C) was used for calibration. The data were analyzed using the provided ASTRA software. The refractive index increment (d*n*/dc) of the polymers was measured by injection of 600 µL of precisely weighted samples in the range of 6 to 15 mg/mL and using a flow rate of 0.6 mL/min in an Optilab Rex detector (Wyatt technology). The results of the d*n*/dc measurements were used to calculate the molecular weight *M*_w(np)_ of the scattering nanoparticles using a Zimm plot and to deduce the aggregation number *N*_agg_ by dividing the *M*_w(np)_ by the weight-averaged molar mass of the polymer chains [48]. Data was analyzed using Astra software.

#### 2.3.3. Cryo-Transmission Electron Microscopy (Cryo-TEM) Analysis

Cryo-TEM analysis on selected samples was performed using a FEI CryoTitan (Thermo Fisher Scientific, Hillsboro, OR, USA) equipped with a field emission gun and autoloader and operated at 300 kV acceleration voltage in low-dose bright-field TEM mode. Samples for cryo-TEM were prepared by glow-discharging the grids (Lacey carbon coated, R2/2, Cu, 200 mesh, EM sciences) in a Cressington 208 carbon coater for 40 s. Then, 4 μL of the nanoparticle dispersion was pipetted onto the grid and blotted in a Vitrobot MARK III at room temperature and 100% humidity. The grid was blotted for 3 s (offset-3) and subsequently frozen in liquid ethane. Cryo-TEM images were acquired with zero loss energy filtering mode (Gatan GIF 2002, 20 eV energy slit) on a charge-coupled device (CCD) camera (Gatan model 794).

#### 2.3.4. Polymer Synthesis

mPEG-*b*-p(HPMA-Bz) block copolymers were synthesized by free radical polymerization as described previously (Supplementary Information, Scheme S1) [42,45,49]. In short, a 4,4-azobis(4-cyanopentanoic acid) (ABCPA) containing macro-initiator, mPEG-ABCPA-mPEG, and HPMA-Bz were dissolved in acetonitrile at varying feed ratios (1:25, 1:50, 1:100, 1:200 mol/mol, respectively). Under a nitrogen atmosphere, the polymerization was conducted at 70 °C for 24 h. The formed polymer was collected by precipitation in excess of ice-cold diethyl ether, followed by filtration and drying under vacuum. The synthesized block copolymers were analyzed by GPC and ^1^H-NMR spectroscopy.

#### 2.3.5. Preparation of Nanoparticles Based on mPEG-b-p(HPMA-Bz) Using Microfluidics

The different mPEG-*b*-p(HPMA-Bz) block copolymers were dissolved in THF (concentrations were 5, 10 and 20 mg/mL) and ultrapure water was used as a non-solvent. Both solutions were filtered prior to use with cellulose acetate 0.22 µm and PTFE 0.22 µm syringe filters, respectively. Pump **A** was filled with ultrapure water and pump **B** with the block copolymer solution in THF. The polymer solution and water were mixed at a 1:1 volume ratio at different total flow rates *Q*_tot_ (100, 200, 350, 500 and 1600 µL/min) and the obtained dispersions were collected at the output into a glass vial until a total volume of 2 mL was obtained. THF was removed by evaporation for 16 h by leaving the vial uncapped in a fume hood, which leads to less than 1 vol% of THF remaining according to our previous study [42]. The formed nanoparticles, prepared in triplicate, were characterized using DLS, AF4-MALLS and cryo-TEM.

## 3. Results and Discussion

### 3.1. Synthesis of mPEG-b-p(HPMA-Bz) Block Copolymers

Amphiphilic mPEG-*b*-p(HPMA-Bz) block copolymers were synthesized through free radical polymerization with varying feed ratios of monomer HPMA-Bz to macro-initiator mPEG-ABCPA-mPEG (M:MI). For all synthesized polymers the yield was approximately 75%. The number- and weight- average molar masses (*M*_n_ and *M*_w_, respectively), the degree of polymerization (*N*_HPMA-Bz_) and the molar mass dispersities (Ð) of the obtained polymers were determined by ^1^H-NMR and GPC analysis (Table 1).

Furthermore, powder mass densities were measured by helium pycnometry. The values for the HPMA-Bz monomer and p(HPMA-Bz) polymer were 1.1796 ± 0.002 and 1.1944 ± 0.0012 g·cm^−3^, respectively. On the other hand, according to the literature, PEG has a mass density of 1.13 g·cm^−3^ [50]. With this information the hydrophilic volume fraction (*Ø*_PEG_) could be estimated (Table 1) by applying the following equation where *f*_PEG_ is the calculated hydrophilic weight fraction, *d*_PEG_ is the mass density of PEG and *d*_p(HPMA-Bz)_ is the mass density of the p(HPMA-Bz) polymer:

(1) ∅PEG=fPEGdPEG[fPEGdPEG+(1−fPEG)dp(HPMA−Bz)]

Interestingly, the volume fractions *Ø*_PEG_ were not very different from the weight fractions *f*_PEG_. Based on the phase diagram reported by Jain and Bates for the low *T*_g_ poly(butadiene)-*b*-poly(ethylene glycol) as a function of the degree of polymerization of the hydrophobic block and the hydrophilic fraction *f*_PEG_ [51], the expected equilibrium morphologies of the self-assemblies were vesicles for block copolymer A (mPEG_5K_-*b*-p(HPMA-Bz)_17.1K_), a blend of vesicles and cylinders for block copolymer B (mPEG_5K_-*b*-p(HPMA-Bz)_10.0K_), only cylinders for block copolymer C (mPEG_5K_-*b*-p(HPMA-Bz)_5.2K_), and spherical micelles for block copolymer D (mPEG_5K_-*b*-p(HPMA-Bz)_2.7K_).

### 3.2. The Effect of Mixing Time on the Size and Morphology of mPEG-b-p(HPMA-Bz) Nanoparticles

The effect on the size and morphology of mPEG-*b*-p(HPMA-Bz) block copolymer nanoparticles formed by the solvent shift method (nanoprecipitation) was studied using microfluidics. By applying total flow rates (*Q*_tot_) ranging from 100 to 1600 µL/min the mixing time $\left(\tau_{M}\right)$ in the micromixer was varied from 1570 to 42 ms according to the data provided by the manufacturer (Supplementary Information, Table S1).

**Figure 2:**
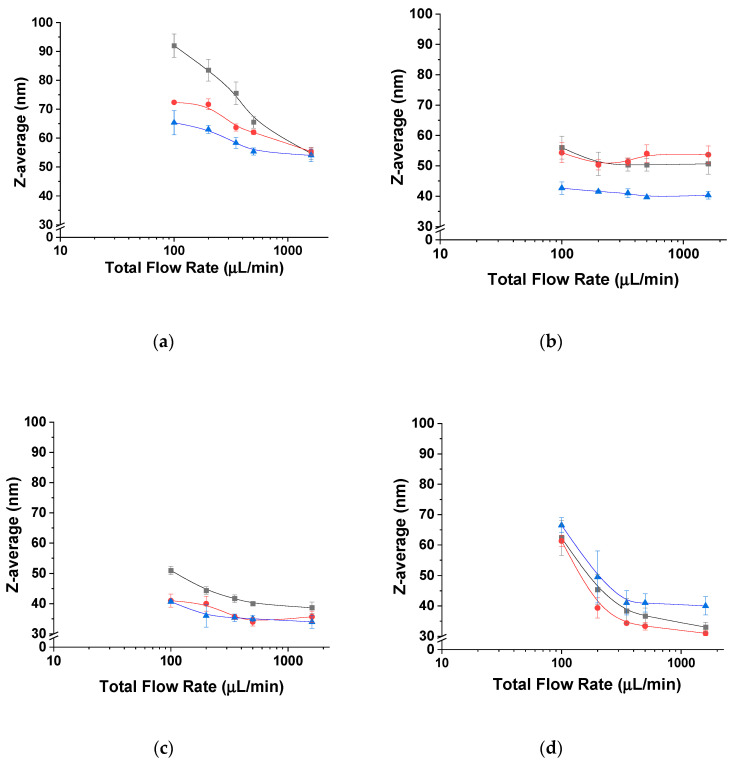
Average hydrodynamic diameter of mPEG_5K_-*b*-p(HPMA-Bz)_X_ nanoparticles as a function of flow rate. (**a**) mPEG_5K_-b-p(HPMA-Bz)_17.1K_, (**b**) mPEG_5K_-*b*-p(HPMA-Bz)_10.0K_, (**c**) mPEG_5K_-*b*-p(HPMA-Bz)_5.2K_ and (**d**) mPEG_5K_-*b*-p(HPMA-Bz)_2.7K_. Black square: 5 mg/mL, red circle: 10 mg/mL and blue triangle: 20 mg/mL block copolymer in THF.

Figure 2a demonstrates that block copolymer A (mPEG_5K_-*b*-p(HPMA-Bz)_17.1K_), with the largest hydrophobic block and lowest *f*_PEG_ (23%), at a polymer concentration of 5 mg/mL assembled into particles increased in size from 55 to 90 nm when the flow rate decreased from 1600 to 100 µL/min. The PDI values for the different nanoparticles were all below 0.2, thereby demonstrating homogeneity in the self-assembly process. Figure 2a also shows that the observed effect was less pronounced upon increasing the concentration of block copolymer A (mPEG_5K_-*b*-p(HPMA-Bz)_17.1K_) to 20 mg/mL. These results can be explained by the nucleation-controlled self-assembly process as the size of the nanoparticle is dependent on the nucleation rate. This is in line with the results from our previous study regarding nanoprecipitation in bulk [42]. In short, the addition of anti-solvent reduces the solubility of block copolymers and induces supersaturation [52]. The nucleation rate is dependent on the supersaturation degree of the block copolymers, which is in turn affected by the used concentration and mixing rate of the polymer-containing solvent and anti-solvent. Slower flow rates result in longer mixing times, which provide a more gradual change in the composition of all the components (solvent, unimers and chain aggregates). This eventually results in less homogeneous supersaturation and slower nucleation and therefore provides a longer growth time of the nanoparticles. Faster flow rates, on the other hand, ensure shorter mixing times. This is associated with rapid and homogenous supersaturation and the formation of more numerous nuclei, which eventually results in smaller and more monodisperse nanoparticles according to the classical nucleation and growth model also called the Lamer model [17].

The Z-average hydrodynamic diameters of self-assemblies based on the block copolymers with larger hydrophilic weight fraction *f*_PEG_, (mPEG_5K_-*b*-p(HPMA-Bz)_10.0K_ (B) and mPEG_5K_-*b*-p(HPMA-Bz)_5.2K_ (C), did not change significantly when different polymer concentrations or flow rates were used (Figure 2b,c). However, self-assembly of block copolymer D with the smallest hydrophobic block and thus the highest *f*_PEG_ (65%), mPEG_5K_-*b*-p(HPMA-Bz)_2.7K_, resulted in an increase in nanoparticle size from 30 to 65 nm upon decreasing the flow rate regardless of the polymer concentration (Figure 2d). Along with an increase in particle size, the PDI values also increased moderately upon decreasing the flow rates (Supplementary Information, Figure S6).

### 3.3. Morphology of mPEG-b-p(HPMA-Bz) nanoparticles

To gain insight into the morphology of the formed nanoparticles based on the largest block copolymer A mPEG_5K_-*b*-p(HPMA-Bz)_17.1K_, the radius of gyration (*R*_g_), hydrodynamic radius (*R*_h_) and size distribution (fractograms) were determined using AF4-MALLS (Table 2). This analytical technique combines the advantages of field-flow fractionation chromatography to separate fractions of nearly monodisperse self-assemblies with the power of multi-angle laser light scattering (MALLS) to get an insight on their morphologies. Interestingly, the *R*_g_/*R*_h_ ratio and the weight average molecular weight of the nanoparticles (*M*_w(np)_) deduced from a Zimm plot gradually increased upon decreasing the flow rate. At the two lower concentrations, 5 and 10 mg/mL, *R*_g_/*R*_h_ ratios of ~1 were observed for the slowest flow rate (100 µL/min) i.e., longest mixing time (1570 ms). However, this was not observed for the highest polymer concentration studied (20 mg/mL) at which *R*_g_/*R*_h_ ratios close to 0.8 were measured at all flow rates.

The *R*_g_/*R*_h_ ratio (or shape factor ρ) is structure sensitive and therefore provides information about the morphology of nanoparticles [53]. In particular, it has been shown that the *R*_g_/*R*_h_ ratios for structures with a dense core and less dense shell (core-shell structures) are lower than 0.775 [54,55,56,57,58]. On the other hand, particles with a rigid spherical structure have in theory *R*_g_/*R*_h_ ratios of ~$\sqrt{3/5}$ or ~0.775 [54,55]. For spherical vesicles like polymersomes, the scattering mass is concentrated on the surface of the sphere yielding a *R*_g_/*R*_h_ ratio near one [59,60]. Therefore, the AF4-MALLS results for block copolymer A (mPEG_5K_-*b*-p(HPMA-Bz)_17.1K_) nanoparticles indicate that polymer vesicles (polymersomes) were formed at slower flow rates, instead of the filled micelles that were formed at higher concentrations and faster flow rates.

**Figure 3:**
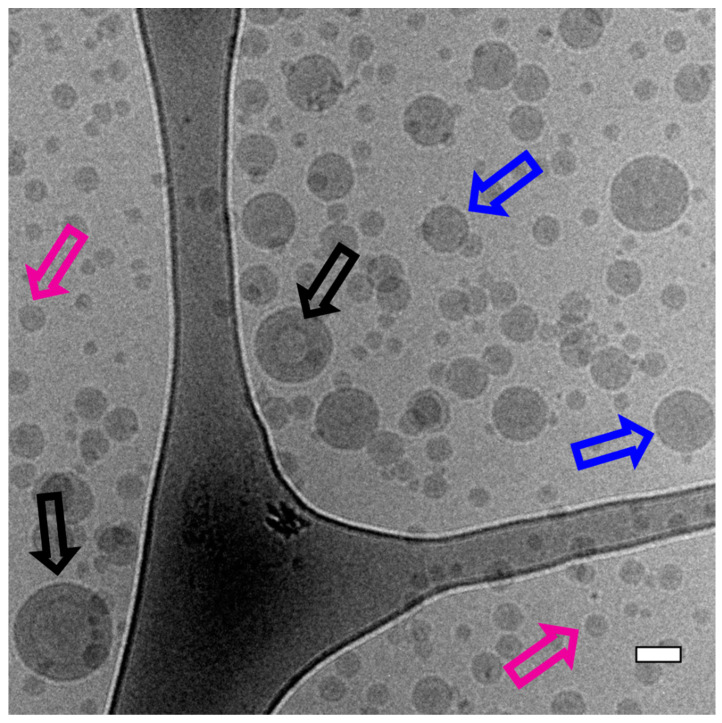
Cryo-TEM overview picture of block copolymer A (mPEG_5K_-*b*-p(HPMA-Bz)_17.1K_) nanoparticles prepared at a concentration of 5 mg/mL and a flow rate of 100 µL/min. Black arrows point to vesicles such as polymersomes, blue arrows point to bigger micelles and the purple arrows point to smaller filled micelles. Scalebar indicates 50 nm.

**Figure 4:**
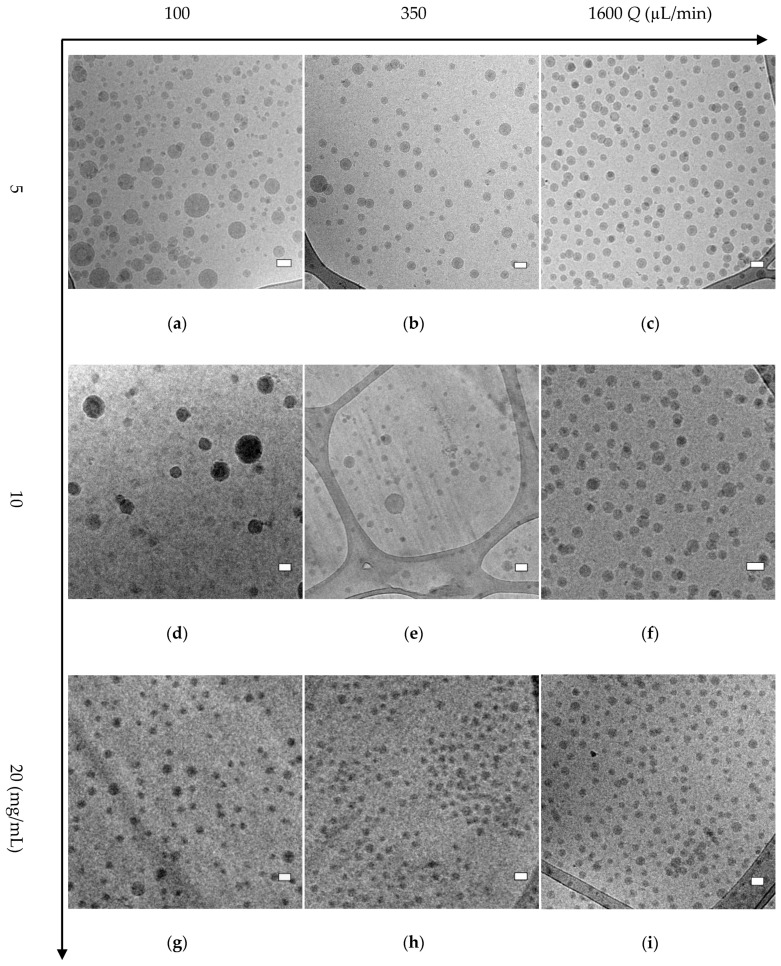
Cryo-TEM pictures of polymer A (mPEG_5K_-*b*-p(HPMA-Bz)_17.1K_) nanoparticles prepared using different polymer concentrations and flow rates. (**a**) 5 mg/mL and 100 µL/min, (**b**) 5 mg/mL and 350 µL/min, (**c**) 5 mg/mL and 1600 µL/min, (**d**) 10 mg/mL and 100 µL/min, (**e**) 10 mg/mL and 350 µL/min, (**f**) 10 mg/mL and 1600 µL/min, (**g**) 20 mg/mL and 100 µL/min, (**h**) 20 mg/mL and 350 µL/min, (**i**) 20 mg/mL and 1600 µL/min. Scale bars indicate 50 nm.

Cryo-TEM analysis of some selected samples was used to corroborate the AF4-MALLS results regarding the nanoparticle morphology of block copolymer A (mPEG_5K_-*b*-p(HPMA-Bz)_17.1K_) nanoparticles. Figure 3 provides an overview of all the observed morphologies. It was shown that for the two lowest concentrations (5 and 10 mg/mL) using slower flow rates, larger micelles and also polymersomes were formed. Interestingly, at the fastest flow rate of 1600 µL/min, regardless of the used concentration, only solid micelles were formed with a diameter of around 35 nm as measured by cryo-TEM (Figure 4). The hydrodynamic diameters for these samples were around 55 nm as measured by DLS (Figure 2a). This apparent discrepancy in diameters can be easily explained. Indeed, cryo-TEM only allows visualization the core of the micelles where the aromatic benzyl groups are localized which provide a high scattering density for electrons, whereas DLS includes the hydrated mPEG corona, which is much transparent to the electron beam. Figure 4 also demonstrates that only micelles were formed at 20 mg/mL, independent of the used flow rates.

**Figure 5:**
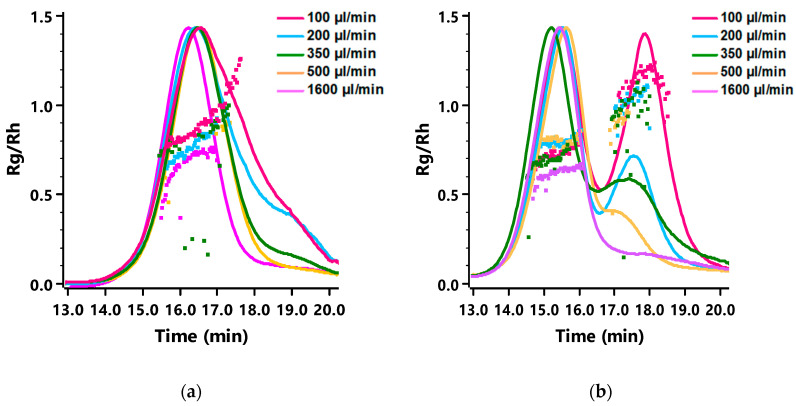
Fractograms of nanoparticles obtained at varying microfluidic flow rates measured with AF4-MALLS. (**a**) Block copolymer A (mPEG_5K_-*b*-p(HPMA-Bz)_17.1K_) at concentration of 5 mg/mL. (**b**) Block copolymer D (mPEG_5K_-*b*-p(HPMA-Bz)_2.7K_) with a concentration of 5 mg/mL.

The fractograms of the AF4-MALLS of the 5 mg/mL samples for block copolymer A revealed only one peak for the particles prepared at the fastest flow rates (500 and 1600 µL/min) and one peak with a tail at higher retention times for particles prepared at microfluidic flow rates below 350 µL/min, which could not be separated even by adjusting the fractionation method (Figure 5a and Supplementary Information Figure S7). This observation is in agreement with the cryo-TEM results, which showed that at slower microfluidic flow rates mostly micelles with a size around 30–35 nm were formed together with some bigger objects of 50–100 nm, presumably micelles and even polymersomes (Figure 4). This transition from homogenous small micelles of 30–35 nm diameter at high microfluidic flow rates to more polydisperse particles where small micelles coexist with larger micelles and vesicles is rather gradual. This explains the tail in the chromatographic fractogram by AF4-MALLS.

AF4-MALLS results of the samples prepared from the smallest block copolymer D (mPEG_5K_-*b*-p(HPMA-Bz)_2.7K_) showed strikingly different fractograms compared to the largest block copolymer A (Figure 5b and Supplementary Information Figure S12). At slower microfluidic flow rates, two distinct peaks corresponding to two populations of nanoparticles were observed, whereas for the shortest mixing time, only one peak and therefore one population was detected.

The *R*_g_ and *R*_h_ of the mPEG_5K_-*b*-p(HPMA-Bz)_2.7K_ nanoparticles were determined for the separate populations by AF4-MALLS (Table 3). Interestingly, the average *R*_g_/*R*_h_ ratios of the nanoparticles of the first peaks were all around 0.7, which points to solid spherical structures (~0.775). On the other hand, the nanoparticles of the second peaks showed higher *R*_g_/*R*_h_ values with some even approaching ~1, suggesting the formation of polymersomes. Moreover, the *M*_w(np)_ of the nanoparticles corresponding to the second peak were considerably higher compared to the first peak, between 10–150 MDa and around 3 MDa, respectively. The results for the first peak are comparable with the values previously reported for micelles from the same polymer prepared in batch mode [42]. These results demonstrate that, independent of polymer concentration, two separate particle populations of very distinct morphologies were formed when flow rates were decreased and thus mixing times increased. The formation of other morphologies was also substantiated by the increasing PDI values as measured by DLS.

**Figure 6:**
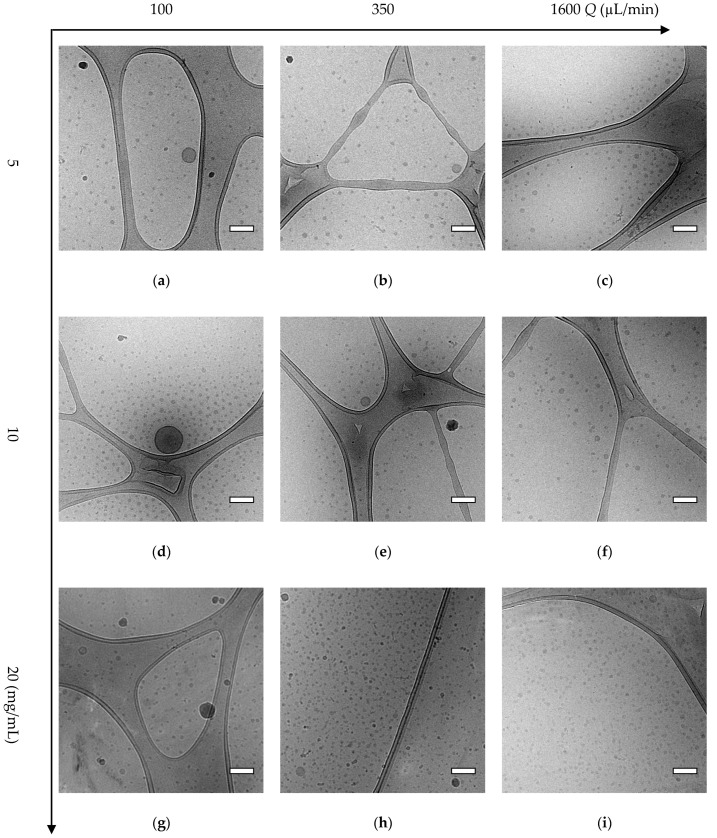
Cryo-TEM pictures of polymer D (mPEG_5K_-*b*-p(HPMA-Bz)_2.7K_) nanoparticles prepared using different polymer concentrations and flow rates. (**a**) 5 mg/mL and 100 µL/min, (**b**) 5 mg/mL and 350 µL/min, (**c**) 5 mg/mL and 1600 µL/min, (**d**) 10 mg/mL and 100 µL/min, (**e**) 10 mg/mL and 350 µL/min, (**f**) 10 mg/mL and 1600 µL/min, (**g**) 20 mg/mL and 100 µL/min, (**h**) 20 mg/mL and 350 µL/min, (**i**) 20 mg/mL and 1600 µL/min. Scale bars indicate 50 nm.

These results are in accordance with the cryo-TEM results of a selection of mPEG_5K_-*b*-p(HPMA-Bz)_2.7K_ nanoparticles (Figure 6). It was shown that mostly small filled micelles with a size around 15–20 nm and a few bigger polymersome structures were formed (Supplementary Information Figure S13).

The *R*_g_/*R*_h_ ratios of block copolymer B (mPEG_5K_-*b*-p(HPMA-Bz)_10.0K_) and C (mPEG_5K_-*b*-p(HPMA-Bz)_5.2K_) nanoparticles showed a main value near 0.775 and a second peak with values between 1.13 and 1.73 for block copolymer B and between 0.92 and 1.38 for block copolymer C (Supplementary Information Tables S2 and S3, Figures S8 and S10), demonstrating that not only solid micelles were formed but also other structures like vesicles depending on the used concentration and flow rate. Cryo-TEM measurements were in accordance with these results and showed that mostly small filled micelles were formed with a size around 30 and 21 nm, respectively, and a few bigger polymersome structures (Supplementary Information Figures S9, S11 and S14).

In the case of mPEG_5K_-*b*-p(HPMA-Bz), with a hydrophobic block of high *T*_g_ (Supplementary Information Figure S15) and aromatic side-groups providing strong ᴨ-ᴨ interactions, nanoprecipitation at fast mixing rates leads to frozen self-assemblies as soon as water and THF are mixed. The occurrence of different morphologies can be explained by the competition between the kinetic process and the thermodynamically favorable structure. Therefore, by using a microfluidic mixing device and performing nanoprecipitation at mixing times *τ*_M_ that could be tuned between 42 and 1570 ms, snapshots of the kinetic process of block copolymer self-assembly were captured.

The mechanism that is best applicable to vesicle formation from mPEG-*b*-p(HPMA-Bz) block copolymers depends on the size of the hydrophobic block. For the largest block copolymer A (mPEG_5K_-*b*-p(HPMA-Bz)_17.1K_) vesicles are expected to be formed at the thermodynamic state, from the packing parameter model with a hydrophilic fraction *f*_PEG_~23% and a hydrophobic block length ***N***_HPMA-Bz_ ~69 [61,62]. It is envisioned that the vesicles are formed through a mechanism as described in detail by He and Schmid [63]. They stated that vesicles form via self-assembly of micelles that subsequently undergo an internal reorganization to yield vesicular membranes. It was shown that, under dilute conditions, first spherical micelles were formed that continue to grow through a path reminiscent of Ostwald ripening of emulsions into larger micelles. These subsequently transform into semi-vesicles through a flip-flop motion of chains that brings the hydrophilic PEG chains inward and drives solvent diffusion inside and eventually reach full vesicle morphologies. The fact that the different sizes and shapes of the particles could not be separated on AF4-MALLS as described above emphasizes a gradual growth of micelles and eventually a rearrangement into lamellar structures. Therefore, this explains why the fractogram of samples prepared from block copolymer A (mPEG_5K_-*b*-p(HPMA-Bz)_17.1K_) at slower microfluidic flow rates was broader and becomes narrower at faster microfluidic flow rates. The cryo-TEM pictures confirmed the proposed mechanism, and all three structures (micelles, larger micelles and vesicles) were observed for particles prepared at the slowest flow rates and the lowest concentration (Figure 3).

From these results, it is apparent that in order to prepare dispersions with only spherical micelles, three factors are important. The first factor is the hydrophobic to hydrophilic ratio, here determined by *f*_PEG_. In this research, it was shown that nanoparticles resulting from all block copolymers resulted mainly into spherical micelles at high concentrations and/or at fast flow rates. This observation is contrary to their equilibrium morphology, which corresponds in theory to vesicles for block copolymer A, a blend of vesicles and of cylindrical (worm-like) micelles for block copolymer B, only cylinders for block copolymer C and spherical micelles for block copolymer D [51]. In this work, vesicles were only detected as small secondary populations at low concentrations and/or slow flow rates, indicating kinetic control of the self-assembly process rather than thermodynamic. The second important factor is the used polymer concentration which determines the supersaturation condition. It was for example observed for block copolymer A (mPEG_5K_-*b*-p(HPMA-Bz)_17.1K_) that nanoprecipitation at high supersaturation condition resulting from using high polymer concentrations is needed in order to obtain spherical micelles only. The third important factor is the flow rate of solvents, or equivalently the mixing time during the nanoprecipitation process, which also has an influence on supersaturation conditions. For both block copolymer A and D (mPEG_5K_-*b*-p(HPMA-Bz)_17.1K_ and mPEG_5K_-*b*-p(HPMA-Bz)_2.7K_, respectively) it was found that higher flow rates led to faster and better mixing and therefore resulted in the formation of micelles only. On the contrary, the lower flow rates led to slower mixing conditions (with mixing time up to 1.6 s), which favors the apparition of self-assemblies with a *R*_g_/*R*_h_-value around one. This is a characteristic of vesicles and was even observed for block copolymer D whose hydrophilic fraction *f*_PEG_~65% and hydrophobic block length ***N***_HPMA-Bz_~11. This indicates a preference for the formation of spherical micelles at thermal equilibrium, according to the classical phase diagram of amphiphilic diblock copolymers [51]. It is hypothesized that vesicle formation proceeds in the case of block copolymer D through a different mechanism. It was proposed that upon mixing a block copolymer solution with a non-solvent for one block, spherical micelles appear first. Then they aggregate through coalescence and grow into larger cylindrical micelles which later fuse into flat membranes that eventually close up on themselves, thereby entrapping solvent to yield vesicles [61,62,64]. Such a scenario of block copolymer self-assembly from micelles to vesicles through cylinders was confirmed with numerical simulation as described by Campos-Villalobos et al. [22]. This is ascribed to a plasticizing effect of THF, enabling chain mobility even at a temperature below the *T*_g_.

In general, for reliable nanoprecipitation of mPEG-*b*-p(HPMA-Bz) block copolymers into spherical micelles, of diameters as small as possible to fit the biological applications, a high nucleation rate should be created. This could be achieved by providing high supersaturation conditions by applying fast mixing rates and using high polymer concentrations. The intrinsic propensity of the block copolymers to form other morphologies, based on their hydrophobic to hydrophilic ratio, was hereby bypassed through the kinetic control. Only at lower mixing rates and lower concentrations these thermodynamically more favorable morphologies became apparent. Finally, after one year, all the samples showed no visible precipitation and evolution when measured again using DLS, indicating that the formed nanoparticle suspensions are stable.

## 4. Conclusions

This study demonstrates that the self-assembly of mPEG-*b*-p(HPMA-Bz) block copolymers into nanoparticles can be easily tailored in size and morphology using microfluidics. This control relies partly on the hydrophobic to hydrophilic ratio of the block copolymers and mostly on the processing methods which change the supersaturation conditions. In general, mPEG-*b*-p(HPMA-Bz) block copolymers formed micelles when both concentration and total flow rate were high. Lowering both concentration and flow rate resulted in a considerable effect on the resulting size and morphology of mPEG-*b*-p(HPMA-Bz) self-assembled nanoparticles. Even polymersomes were formed for block copolymers which supposedly self-assemble into spherical micelles at the thermodynamic state. However, other time-resolved experiments such X-ray or neutron scattering techniques would be necessary to definitively describe the pathway from unimers to self-assemblies. Importantly, microfluidics is a very suitable method to prepare micelles in a scalable and reproducible manner. For future scaled-up work, using microfluidics is preferred over batch-wise production as it offers more control over the size and morphology of the nanoparticles that are produced.

## Figures and Tables

**Table 1:** Characteristics of the synthesized poly(ethylene glycol)-*b*-poly(*N*-2-benzoyloxypropyl methacrylamide) mPEG_5K_-*b*-p(HPMA-Bz)_x_ block copolymers as determined by ^1^H-NMR and gel permeation chromatography (GPC).

Polymer	M:MI	*M*_n_	GPC			*N*_HPMA-Bz_	*f*_PEG_	*Ø*_PEG_
			*M*_n_	*M*_w_	Ð			
A: mPEG_5K_-*b*-p(HPMA-Bz)_17.1K_	200	22.1	15.8	20.7	1.31	69	23	24
B: mPEG_5K_-*b*-p(HPMA-Bz)_10.0K_	100	15.0	13.2	17.5	1.32	40	33	35
C: mPEG_5K_-*b*-p(HPMA-Bz)_5.2K_	50	10.2	10.8	14.0	1.30	21	49	50
D: mPEG_5K_-*b*-p(HPMA-Bz)_2.7K_	25	7.7	8.9	11.0	1.24	11	65	66

M:MI, monomer to macro-initiator ratio (mol/mol); *M*_n_, number average molar mass (kDa); *M*_w_, weight average molar mass (kDa); Ð, molar mass dispersity; *N*_HPMA-Bz_, degree of polymerization of HPMA-Bz; *f*_PEG_, hydrophilic weight fraction (wt%) and *Ø*_PEG_, hydrophilic volume fraction (vol%).

**Table 2:** Characteristics of block copolymer A (mPEG_5K_-*b*-p(HPMA-Bz)_17.1K_) nanoparticles as determined by asymmetric flow field-flow fractionation connected to multi-angle laser light scattering detector (AF4-MALLS).

Concentration (mg/mL)	Q (µL/min)	*R*_g_ (nm)	*R*_h_ (nm)	*R*_g_/*R*_h_	*M*_w(np)_ (10^3^ kDa)	*N*_agg_
5	100	46	45	1.03	187	8500
5	200	35	39	0.90	142	6400
5	350	32	36	0.89	131	5900
5	500	30	37	0.82	150	6800
5	1600	21	26	0.81	36	1600
10	100	34	33	1.03	79	3600
10	200	24	30	0.82	64	2900
10	350	24	28	0.86	42	1900
10	500	22	28	0.78	39	1800
10	1600	17	25	0.69	26	1200
20	100	24	28	0.85	42	1900
20	200	22	26	0.82	34	1600
20	350	20	27	0.73	69	3100
20	500	21	28	0.76	36	1600
20	1600	20	25	0.78	34	1500

*Q*, flow rate; *R*_g_, radius of gyration; *R*_h_, hydrodynamic radius; *M*_w(np)_, weight average molecular weight of the nanoparticles and *N*_agg_, nanoparticle aggregation number.

**Table 3:** Characteristics of polymer D (mPEG_5K_-*b*-p(HPMA-Bz)_2.7K_) nanoparticles as determined by AF4-MALLS.

Concentration (mg/mL)	Q (µL/min)	Peak 1					Peak 2				
		*R*_g_ (nm)	*R*_h_ (nm)	*R*_g_/*R*_h_	*M*_w(np)_ (10^3^ kDa)	*N*_agg_	*R*_g_ (nm)	*R*_h_ (nm)	*R*_g_/*R*_h_	*M*_w(np)_ (10^3^ kDa)	*N*_agg_
5	100	13	17	0.76	3.1	400	54	54	0.99	98	12,700
5	200	13	17	0.80	3.2	420	46	51	0.91	68	8800
5	350	13	17	0.77	3.3	430	34	40	0.85	91	11,800
5	500	12	17	0.68	3.5	450	32	39	0.82	171	22,200
5	1600	11	17	0.63	3.4	450	-	-	-	-	-
10	100	10	16	0.65	2.5	320	53	56	0.93	70	9000
10	200	11	16	0.67	2.9	380	47	76	0.62	11	1500
10	350	11	17	0.63	3.2	420	26	39	0.65	625	81,200
10	500	12	16	0.71	2.6	340	-	39	-	-	-
10	1600	13	17	0.77	2.8	360	-	-	-	-	-
20	100	12	16	0.75	2.3	300	59	54	1.09	147	19,100
20	200	13	16	0.81	2.3	300	47	45	1.04	145	18,780
20	350	11	16	0.71	2.5	320	48	45	1.06	66	8500
20	500	13	16	0.81	2.3	300	31	36	0.87	104	13,600
20	1600	11	16	0.75	2.5	320	-	-	-	-	-

*Q*, flow rate; *R*_g_, radius of gyration; *R*_h_, hydrodynamic radius; *M*_w(np)_, weight average molecular weight of the nanoparticles and *N*_agg_, nanoparticle aggregation number.

## Supplementary Materials

The following are available online at https://www.mdpi.com/2073-4360/12/11/2572/s1, Scheme S1: Synthesis of mPEG-*b*-p(HPMA-Bz), Figure S1: ^1^H-NMR of block copolymer A mPEG_5K_-b-p(HPMA-Bz)_17.1K_, Figure S2: ^1^H-NMR of block copolymer B mPEG_5K_-*b*-p(HPMA-Bz)_10.0K_, Figure S3: ^1^H-NMR of block copolymer C mPEG_5K_-*b*-p(HPMA-Bz)_5.2K_, Figure S4: ^1^H-NMR of block copolymer D mPEG_5K_-*b*-p(HPMA-Bz)_2.7K_, Figure S5: Mixing time (ms) of NaOH and phenolphthalein solutions plotted against total flowrates, Table S1: Flow rates and their approximated mixing times as calculated using the information from Figure S5, Figure S6: PDI values of block copolymer D mPEG_5K_-*b*-p(HPMA-Bz)_2.7K_ nanostructures as a function of mixing time, Figure S7: Rg/Rh traces of the AF4-MALS fractograms of nanoparticles made with block copolymer A mPEG_5K_-*b*-p(HPMA-Bz)_17.1K_ with a concentration of 5 mg/mL at different microfluidic flow rates, Table S2: Characteristics of block copolymer B mPEG_5K_-*b*-p(HPMA-Bz)_10.0K_ nanoparticles as determined by AF4-MALLS, Figure S8: Rg/Rh traces of the AF4-MALS fractograms of nanoparticles made with block copolymer B mPEG_5K_-*b*-p(HPMA-Bz)_10.0K_, Figure S9: Cryo-TEM pictures of block copolymer B mPEG_5K_-*b*-p(HPMA-Bz)_10.0K_ nanoparticles at different flow rates, Table S3: Characteristics of block copolymer C mPEG_5K_-*b*-p(HPMA-Bz)_5.2K_ nanoparticles as determined by AF4-MALLS, Figure S10: Rg/Rh traces of the AF4-MALS fractograms of nanoparticles made with block copolymer C mPEG_5K_-*b*-p(HPMA-Bz)_5.2K_, Figure S11: Cryo-TEM pictures of block copolymer C mPEG_5K_-*b*-p(HPMA-Bz)_5.2K_ nanoparticles, Figure S12: Rg/Rh traces of the AF4-MALS fractograms of nanoparticles made with block copolymer D mPEG_5K_-*b*-p(HPMA-Bz)_2.7K_, Figure S13: Histograms of cryo-TEM diameters of block copolymer A (mPEG_5K_-*b*-p(HPMA-Bz)_17.1K_) and block copolymer D (mPEG*5K*-*b*-p(HPMA-Bz)_2.7K_) nanostructures, Figure S14: Histograms of cryo-TEM diameters of block copolymer B (mPEG_5K_-*b*-p(HPMA-Bz)_10.0K_) and block copolymer C (mPEG_5K_-*b*-p(HPMA-Bz)_5.2K_) nanostructures, Figure S15: Thermograms of p(HPMA-Bz) homopolymers corresponding to the different molecular weight block copolymers recorded by DSC.
