# Supplementary material for: Tuning Size and Morphology of mPEG-b-p(HPMA-Bz) Copolymer Self-Assemblies Using Microfluidics
Source: Polymers (Basel). 2020 Nov 2;12(11):2572. doi: 10.3390/polym12112572 (PMC7693845; doi:10.3390/polym12112572)
Supplement: Supplementary file 1 [file polymers-12-02572-s001.pdf]

polymers

Supporting information

Tuning size and morphology of mPEG-*b*-p(HPMA-Bz) copolymer self-assemblies using microfluidics

Jaleesa Bresseleers [1,2,†], Mahsa Bagheri [3,†], Coralie Lebleu [4], Sébastien Lecommandoux [4], Olivier Sandre [4], Imke A. B. Pijpers [1], Alexander F. Mason [1], Silvie A. Meeuwissen [2], Cornelus F. van Nostrum [3], Wim E. Hennink [3] and Jan C.M. van Hest [1,*]

[1] Department of Bio-Organic chemistry, Eindhoven University of Technology, 5600 MB Eindhoven, the Netherlands; j.bresseleers@tue.nl (J.B.); i.a.b.pijpers@tue.nl (I.A.B.P.); a.f.mason@tue.nl (A.F.M.); j.c.m.v.hest@tue.nl (J.C.M.v.H.)
[2] Ardena Oss, 5349 AB Oss, the Netherlands; silvie.meeuwissen@ardena.com (S.A.M.)
[3] Department of Pharmaceutics, Utrecht Institute for Pharmaceutical Sciences (UIPS), Faculty of Science, Utrecht University, 3508 TB Utrecht, the Netherlands; m.bagheri@uu.nl (M.B.); c.f.vannostrum@uu.nl (C.F.v.N.); w.e.hennink@uu.nl (W.E.H.)
[4] Laboratoire de Chimie de Polymères Organiques, Université de Bordeaux, UMR 5629 CNRS, Bordeaux-INP, 33600 Pessac, France; Coralie.Lebleu@enscbp.fr (C.L.); Lecommandoux@enscbp.fr (S.L.); olivier.sandre@enscbp.fr (O.S.)

* Correspondence: J.C.M.v.Hest@tue.nl; Tel: +31 40 247 3515

† These authors contributed equally to this work.

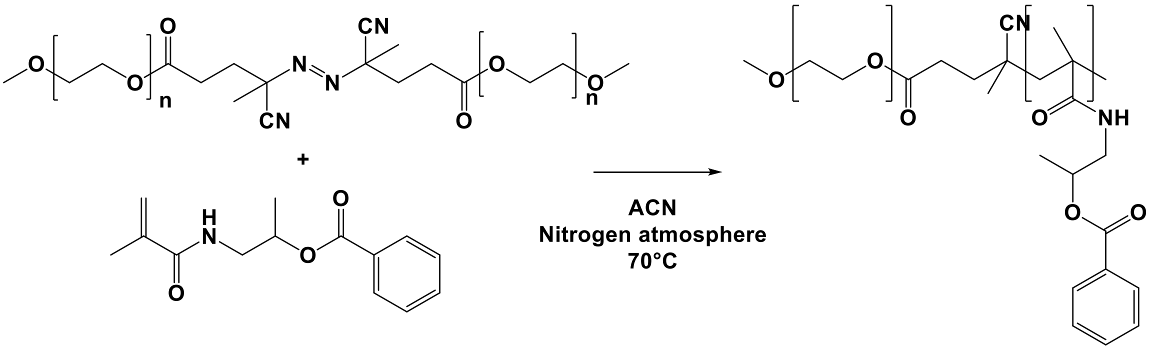

Scheme S1. Synthesis of mPEG-*b*-p(HPMA-Bz).

^{1}H-NMR of mPEG-*b*-p(HPMA-Bz): 8.0 (b, 2H, aromatic CH), 7.55 (b, 1H, aromatic CH), 7.65 (b, 2H, aromatic CH), 7.35 (b, CO-N<u>H</u>-CH_2), 5.0 (b, NH-CH_2-C<u>H</u>(CH_3)-O-(Bz)), 3.40–3.60 (b, mPEG5000 methylene protons, O-C<u>H</u>$_2$-C<u>H</u>$_2$), 3.1 (b, NH-CH_2-CH), 0.6–2.2 (b, the rest of the protons are from the methyl and backbone CH_2 protons).

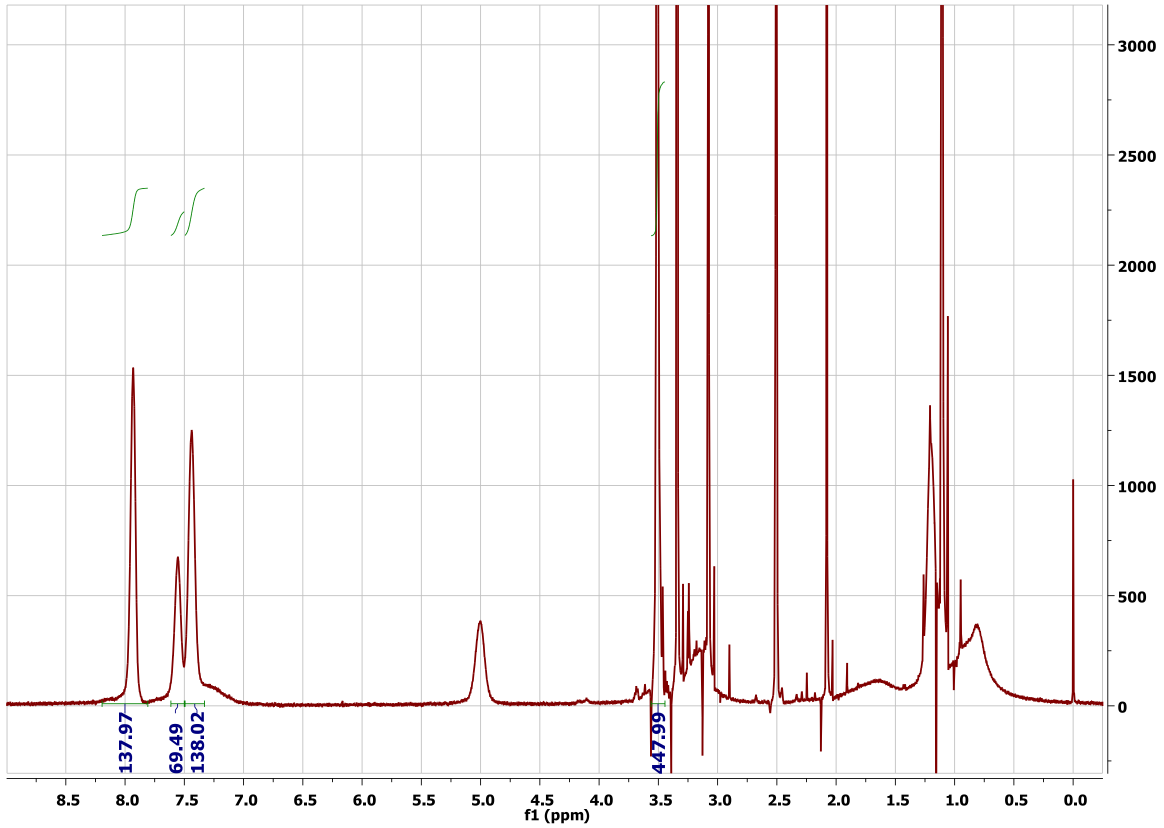

Figure S1. ^{1}H-NMR of block copolymer A mPEG$_{5K}$-b-p(HPMA-Bz)$_{17.1K}$.

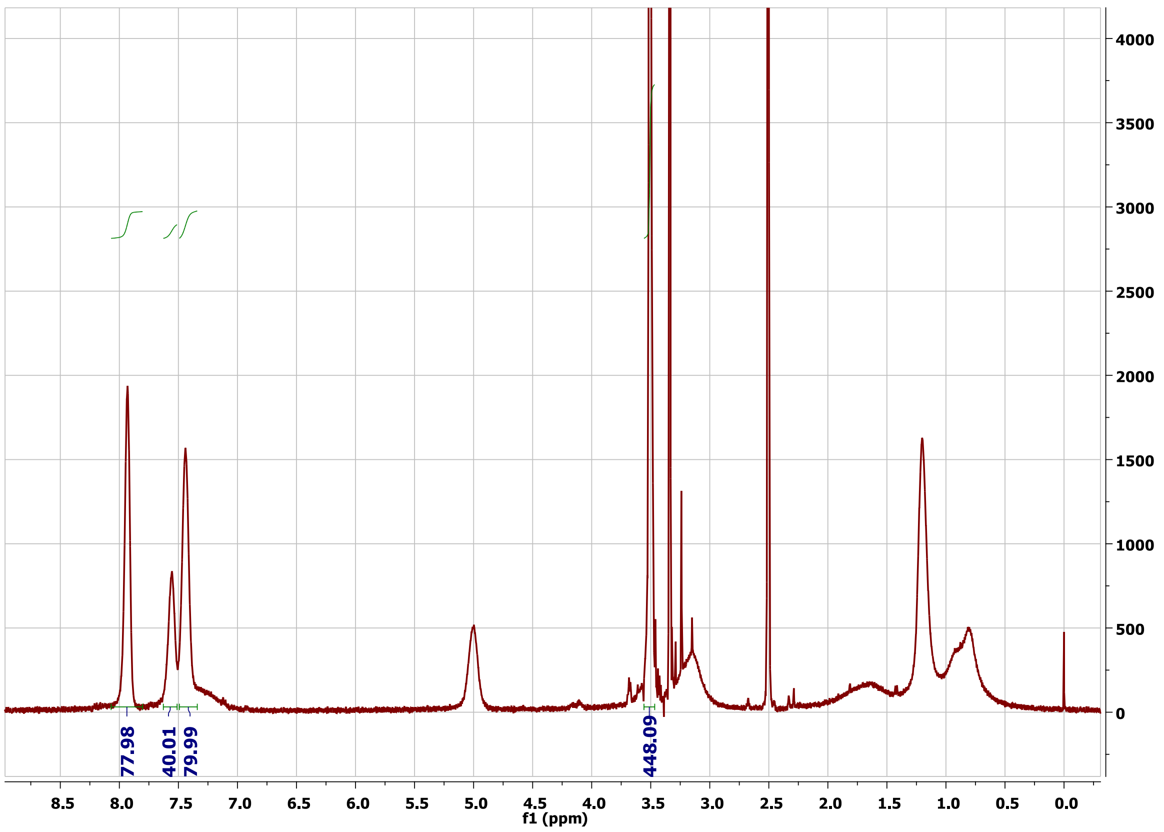

30 **Figure S2.** ^{1}H-NMR of block copolymer B mPEG$_{5K}$-*b*-p(HPMA-Bz)$_{10.0K}$.

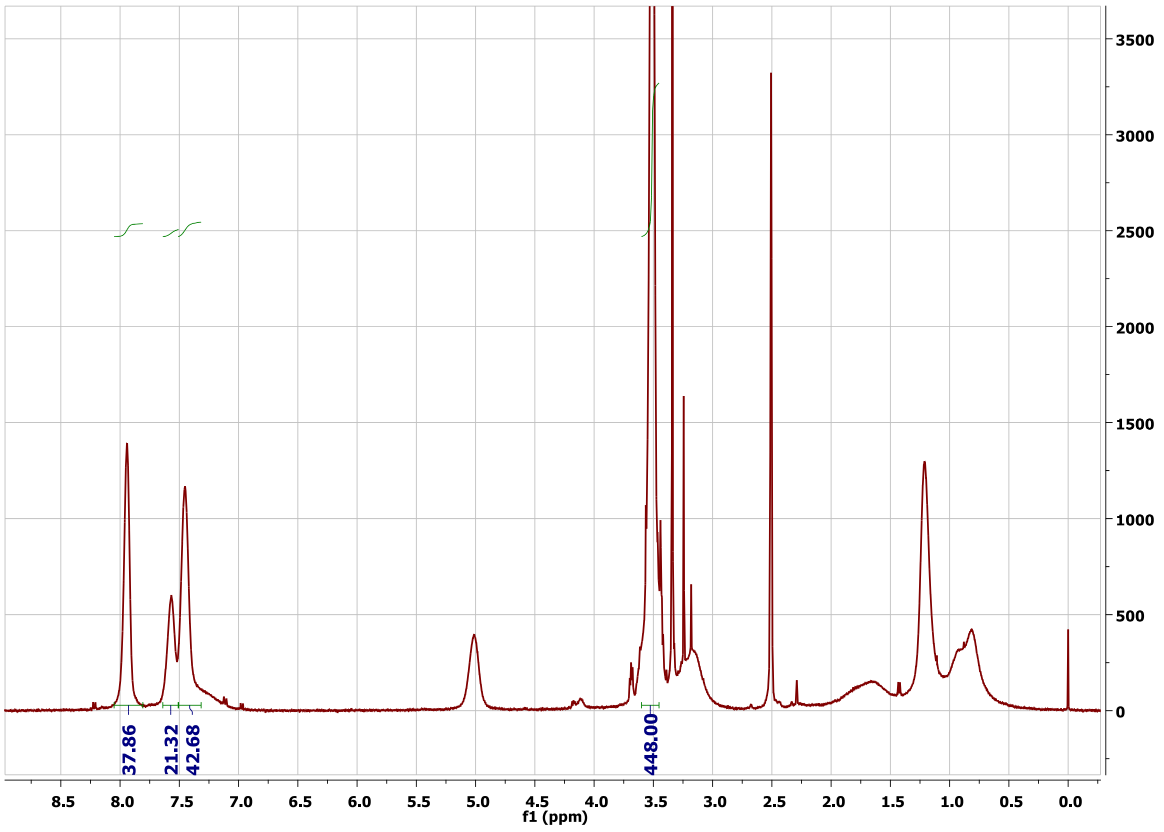

31 **Figure S3.** ^{1}H-NMR of block copolymer C mPEG$_{5K}$-*b*-p(HPMA-Bz)$_{5.2K}$.

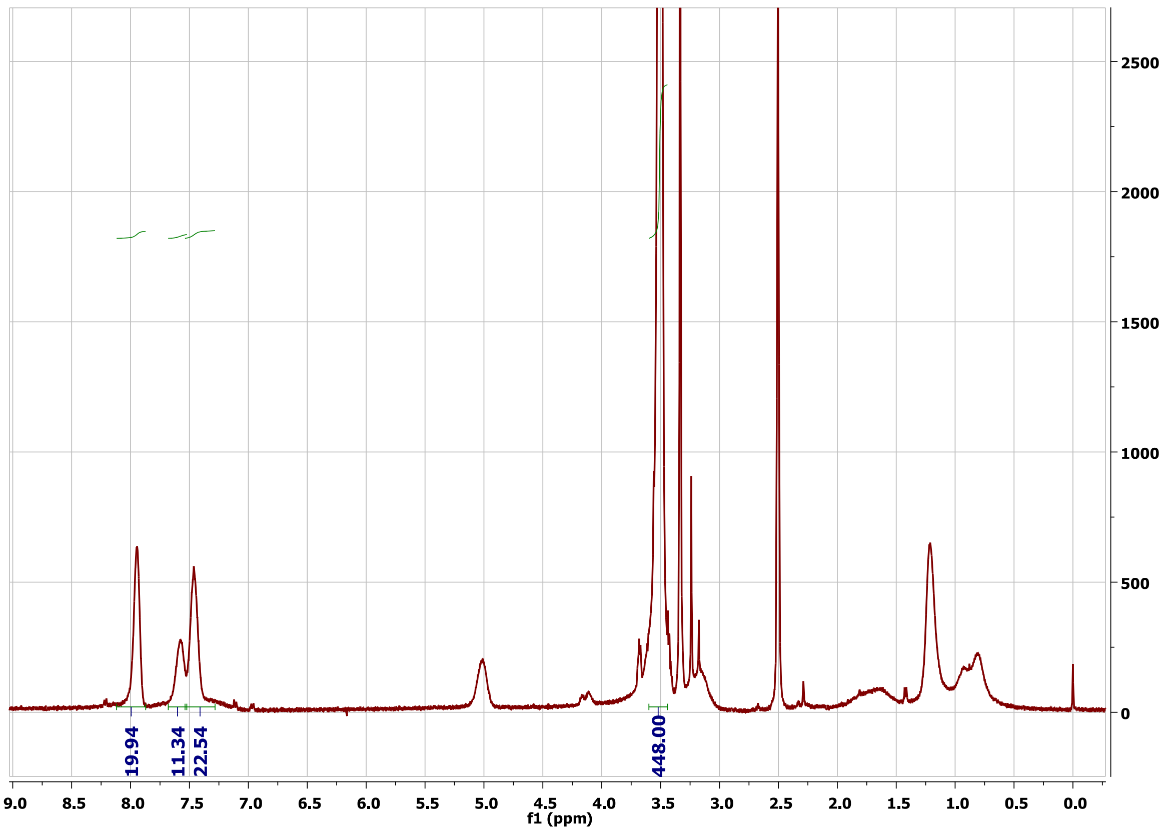

Figure S4. ^{1}H-NMR of block copolymer D mPEG$_{5K}$-*b*-p(HPMA-Bz)$_{2.7K}$.

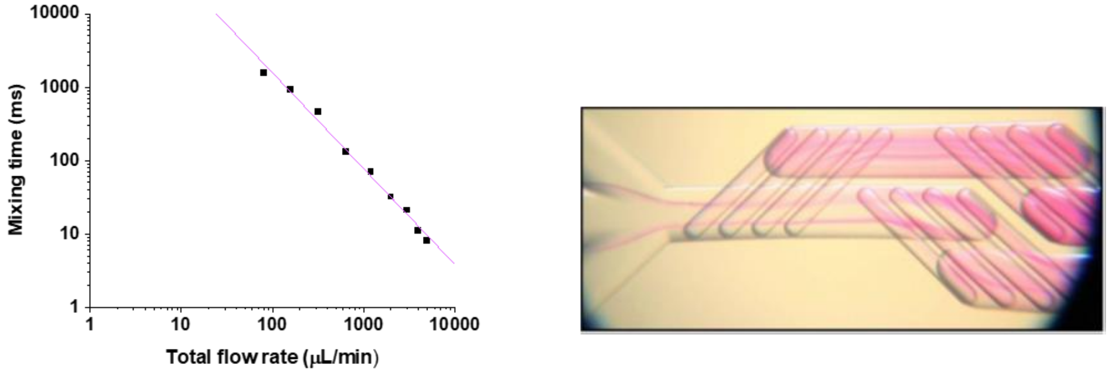

(**a**) (**b**)

Figure S5. (a) Mixing time τ_M (ms) of NaOH and phenolphthalein solutions plotted against total flowrates Q_{tot} (μL/min) for 1:1 ratio at each pump and extrapolated to the following equation $(\tau_M) = 6.4133 \cdot 10^4\, Q_{tot}^{1.306}$. (b) The photograph shows the calibration experiment of the mixing time using two identical flowrates of respectively phenolphthalein and NaOH solutions. Data and photograph were taken from the specifications on the manufacturer's website [1].

Table S1. Flow rates and their approximated mixing times as calculated using the information from **Figure S5**.

Q_{tot} (μL/min)	τ_M (ms)
100	1570
200	634
350	305
500	192
1600	42

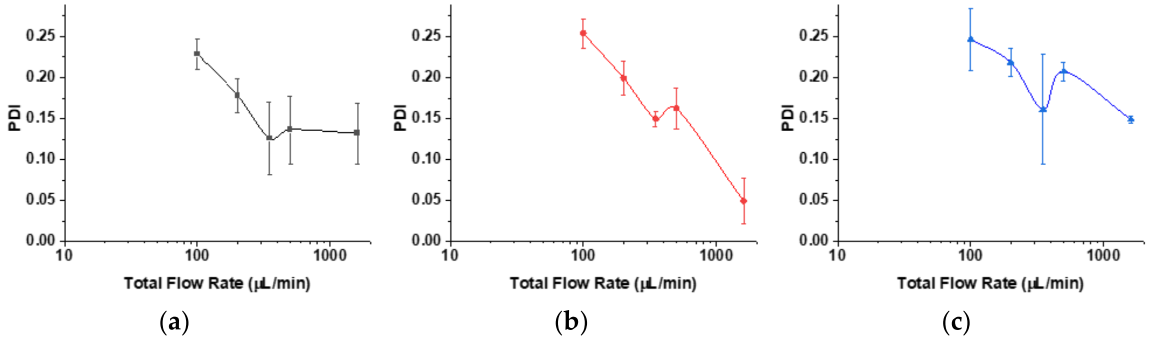

Figure S6. PDI values of block copolymer D mPEG$_{5K}$-*b*-p(HPMA-Bz)$_{2.7K}$ nanostructures as a function of mixing time. (**a**) 5 mg/mL, (**b**) 10 mg/mL and (**c**) 20 mg/mL.

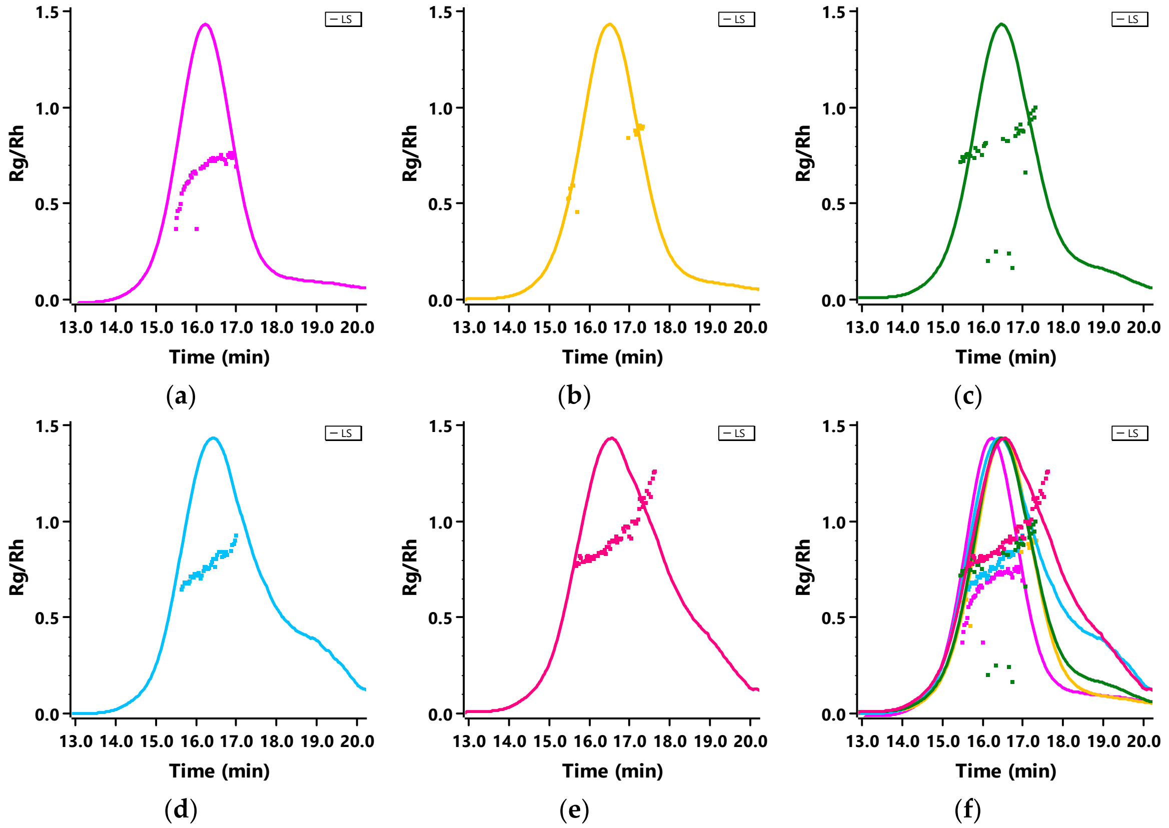

Figure S7. R_g/R_h traces of the AF4-MALS fractograms of nanoparticles made with block copolymer A mPEG$_{5K}$-b-p(HPMA-Bz)$_{17.1K}$ with a concentration of 5 mg/mL and microfluidic flow rates (a) 1600 μL/min, (b) 500 μL/min, (c) 350 μL/min, (d) 200 μL/min, (e) 100 μL/min and (f) all microfluidic flow rates together in one graph.

Table S2. Characteristics of block copolymer B mPEG$_{5K}$-*b*-p(HPMA-Bz)$_{10.0K}$ nanoparticles as determined by AF4-MALLS.

Concentration (mg/mL)	Q (µL/min)	Peak 1					Peak 2				
		R_g (nm)	R_h (nm)	R_g/R_h	$M_{w(np)}$ (10^3 kDa)	N_{agg}	R_g (nm)	R_h (nm)	R_g/R_h	$M_{w(np)}$ (10^3 kDa)	N_{agg}
5	100	19	25	0.77	16	1060	-	-	-	-	-
5	200	18	25	0.72	16	1060	-	-	-	-	-
5	350	17	23	0.72	15	1000	-	-	-	-	-
5	500	14	22	0.63	13	880	73	49	1.49	232	15500
5	1600	13	22	0.59	13	860	84	52	1.62	263	17500
10	100	15	21	0.72	12	770	52	46	1.13	1263	84200
10	200	13	20	0.65	11	720	70	46	1.52	585	39000
10	350	14	20	0.69	11	730	80	48	1.67	1717	114500
10	500	14	21	0.69	12	780	-	51	-	-	-
10	1600	14	20	0.67	11	730	85	49	1.73	1441	96100
20	100	14	20	0.69	9.6	640	-	-	-	-	-
20	200	16	21	0.75	9.9	660	-	-	-	-	-
20	350	14	20	0.70	9.6	640	-	-	-	-	-
20	500	11	20	0.56	9.9	660	-	-	-	-	-
20	1600	13	20	0.65	9.9	660	-	-	-	-	-

Q, flow rate; R_g, radius of gyration; R_h, hydrodynamic radius; $M_{w(np)}$, weight average molecular weight of the nanoparticles and N_{agg}, nanoparticle aggregation number.

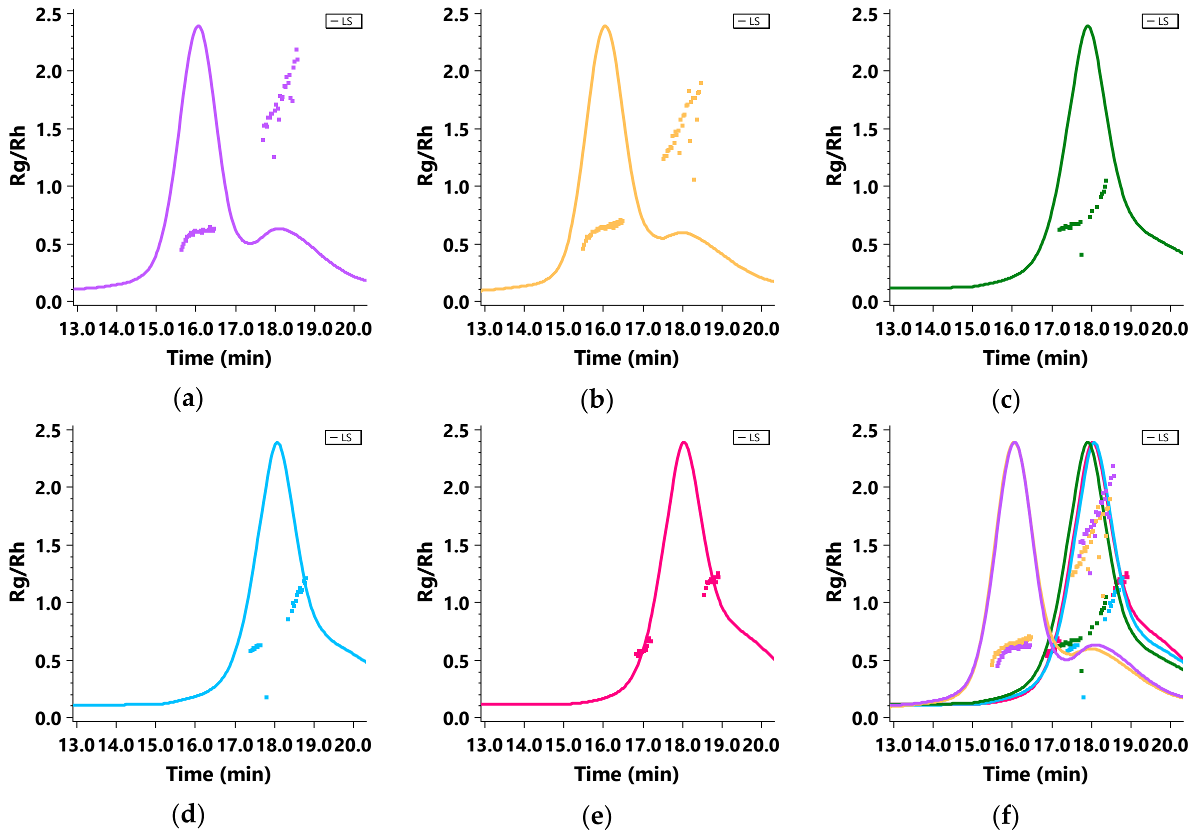

Figure S8. R_g/R_h traces of the AF4-MALS fractograms of nanoparticles made with block copolymer B mPEG$_{5K}$-*b*-p(HPMA-Bz)$_{10.0K}$ with a concentration of 5 mg/mL and microfluidic flow rates (a) 1600 µL/min, (b) 500 µL/min, (c) 350 µL/min, (d) 200 µL/min, (e) 100 µL/min and (f) all microfluidic flow rates together in one graph.

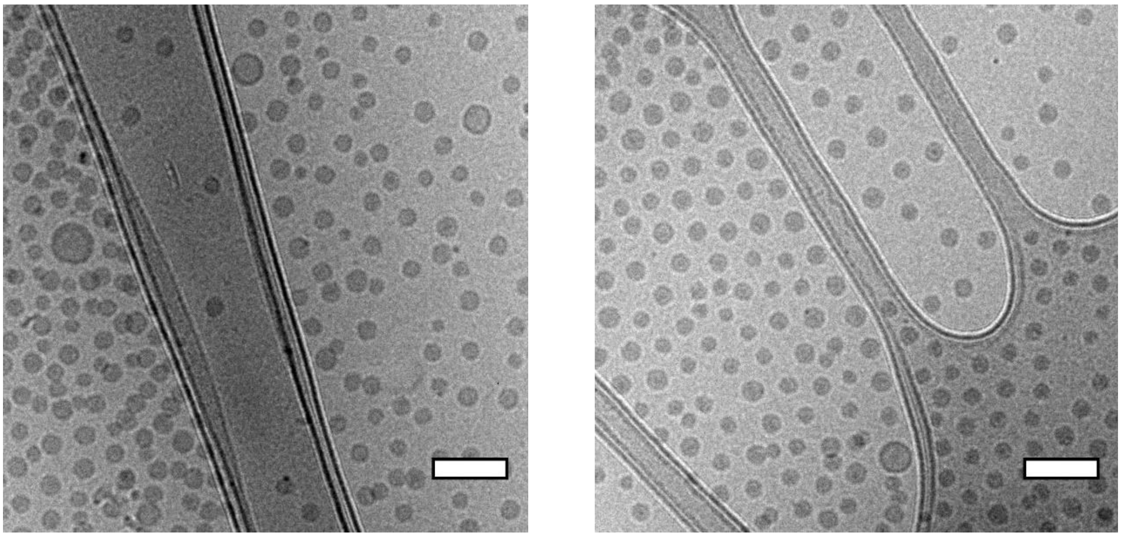

(**a**) (**b**)

Figure S9. Cryo-TEM pictures of block copolymer B mPEG$_{5K}$-*b*-p(HPMA-Bz)$_{10.0K}$ nanoparticles prepared at a concentration of 10 mg/mL and flow rates Scale bars indicate 100 nm. (a) 100 µL/min and (b) 350 µL/min.

Table S3. Characteristics of polymer C mPEG$_{5K}$-*b*-p(HPMA-Bz)$_{5.2K}$ nanoparticles as determined by AF4-MALLS.

		Peak 1					Peak 2				
**Concentration (mg/mL)**	**Q (µL/min)**	**R_g (nm)**	**R_h (nm)**	**R_g/R_h**	**$M_{w(np)}$ (10³ kDa)**	**N_{agg}**	**R_g (nm)**	**R_h (nm)**	**R_g/R_h**	**$M_{w(np)}$ (10³ kDa)**	**N_{agg}**
5	100	12	20	0.61	6.5	640	-	-	-	-	-
5	200	11	18	0.63	5.2	510	41	44	0.93	96	9400
5	350	11	18	0.64	5.3	520	35	38	0.92	48	4700
5	500	11	19	0.56	5.9	580	-	-	-	-	-
5	1600	11	18	0.65	5.3	520	-	-	-	-	-
10	100	14	17	0.80	4.5	440	58	42	1.38	210	20200
10	200	13	17	0.77	4.7	460	-	-	-	-	-
10	350	11	17	0.66	4.3	420	-	-	-	-	-
10	500	13	17	0.76	4.3	420	-	-	-	-	-
10	1600	12	17	0.72	4.6	450	-	-	-	-	-
20	100	12	17	0.73	4.1	400	87	116	0.75	25	2500
20	200	10	16	0.60	3.6	360	-	-	-	-	-
20	350	11	16	0.68	3.9	380	-	-	-	-	-
20	500	11	17	0.67	3.9	390	-	-	-	-	-
20	1600	10	16	0.63	3.6	360	-	-	-	-	-

Q, flow rate; R_g, radius of gyration; R_h, hydrodynamic radius; $M_{w(np)}$, weight average molecular weight of the nanoparticles and N_{agg}, nanoparticle aggregation number.

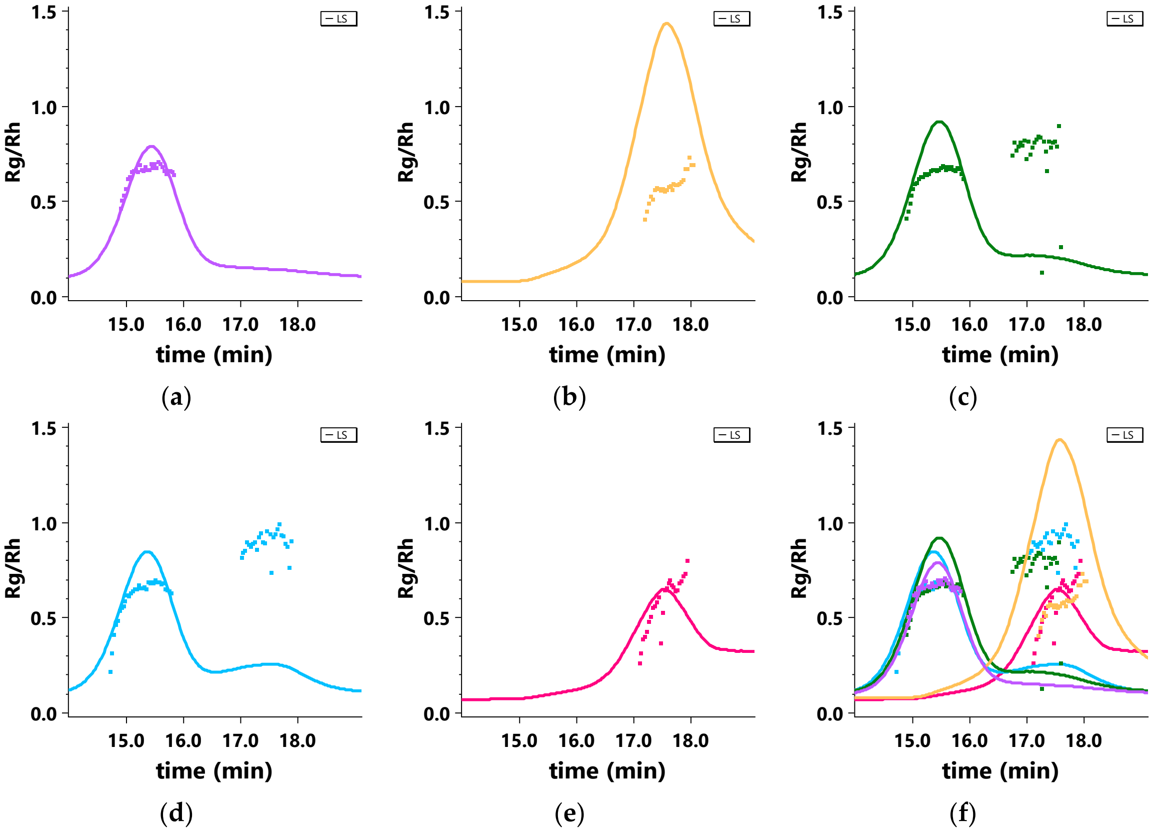

Figure S10. R_g/R_h traces of the AF4-MALS fractograms of nanoparticles made with block copolymer C mPEG$_{5K}$-*b*-p(HPMA-Bz)$_{5.2K}$ with a concentration of 5 mg/mL and microfluidic flow rates (a) 1600 µL/min, (b) 500 µL/min, (c) 350 µL/min, (d) 200 µL/min, (e) 100 µL/min and (f) all microfluidic flow rates together in one graph.

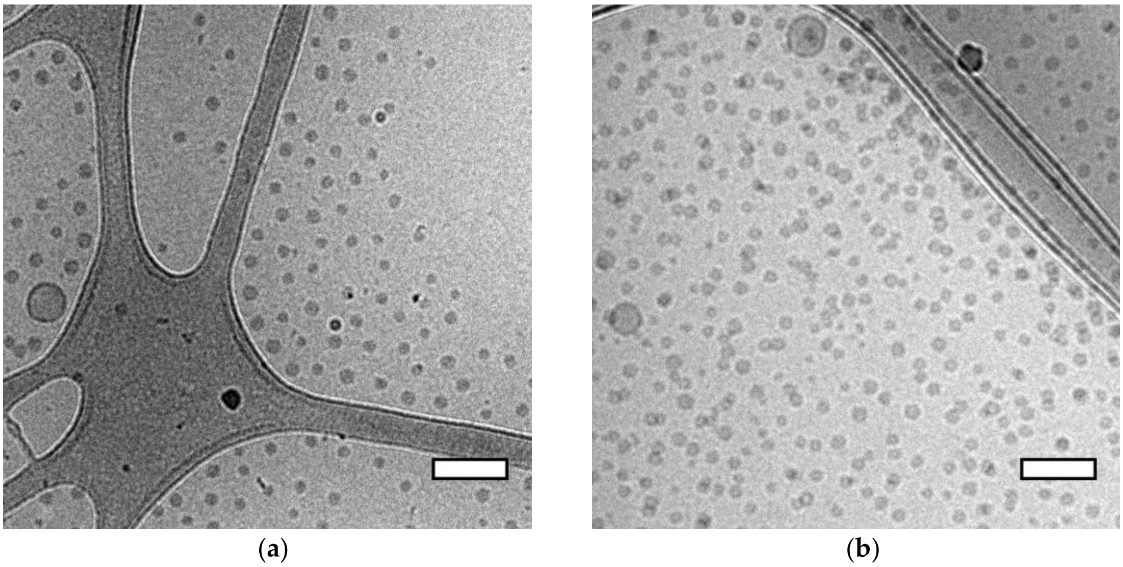

Figure S11. Cryo-TEM pictures of block copolymer C mPEG$_{5K}$-*b*-p(HPMA-Bz)$_{5.2K}$ nanoparticles prepared at a concentration of 5 mg/mL and flow rates. Scale bars indicate 100 nm. (a) 100 µL/min and (b) 350 µL/min.

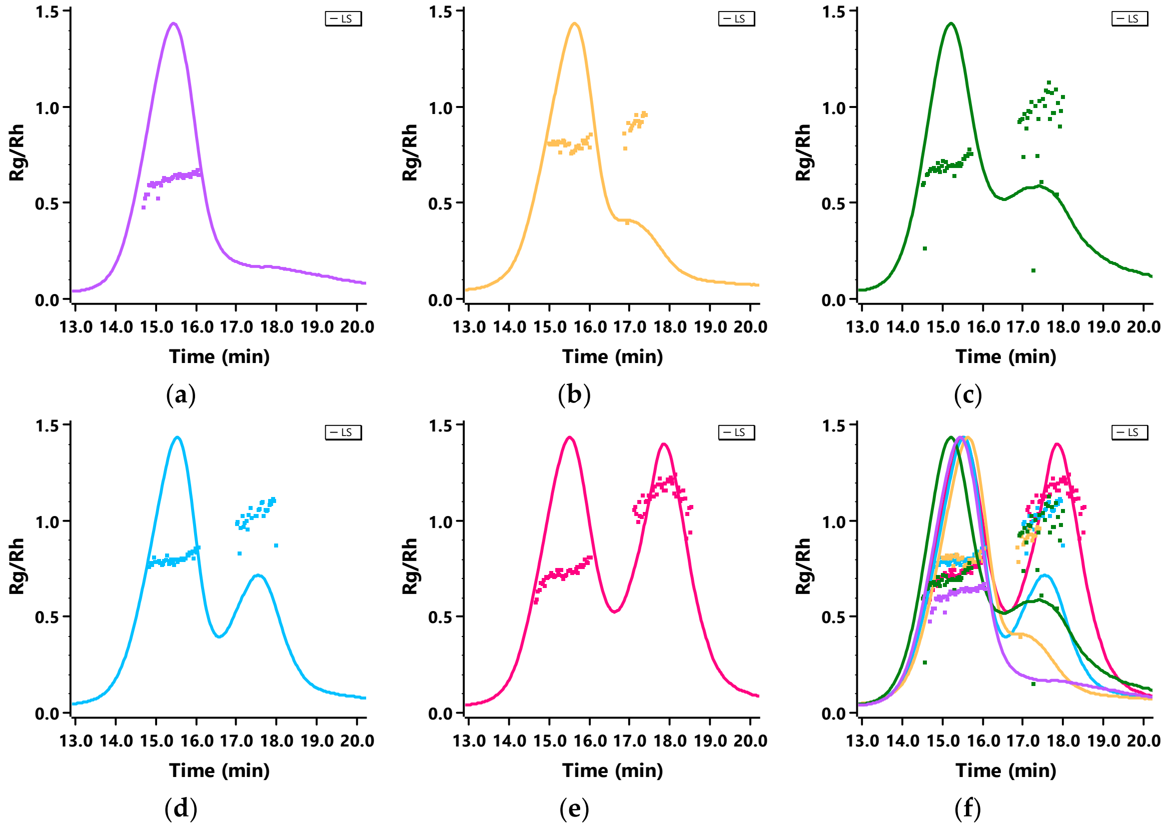

Figure S12. R_g/R_h traces of the AF4-MALS fractograms of nanoparticles made with block copolymer D mPEG$_{5K}$-*b*-p(HPMA-Bz)$_{2.7K}$ with a concentration of 5 mg/mL and microfluidic flow rates (a) 1600 µL/min, (b) 500 µL/min, (c) 350 µL/min, (d) 200 µL/min, (e) 100 µL/min and (f) all microfluidic flow rates together in one graph.

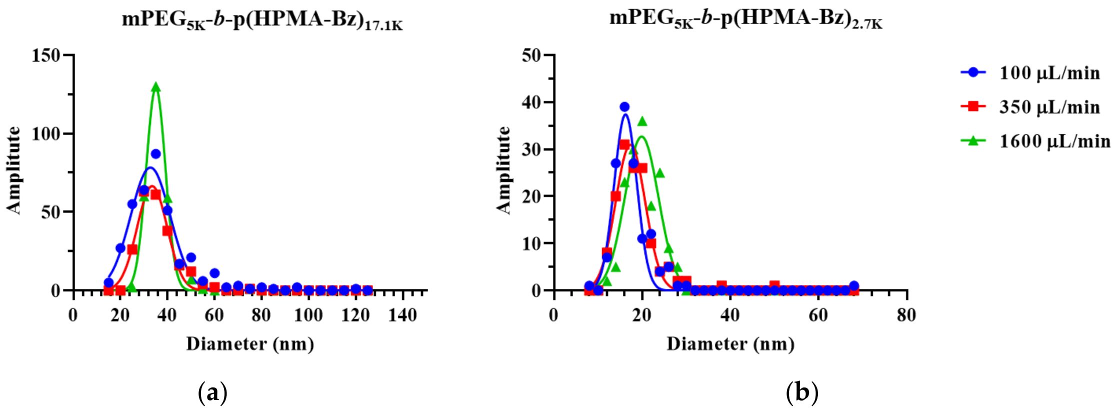

Figure S13. Histograms of cryo-TEM diameters of (a) block copolymer A (mPEG$_{5K}$-b-p(HPMA-Bz)$_{17.1K}$) and (b) block copolymer D (mPEG$_{5K}$-b-p(HPMA-Bz)$_{2.7K}$) nanostructures prepared at 5 mg/ml polymer concentration and different flow rates. The data are fitted by Gaussian laws using GraphPad Prism.

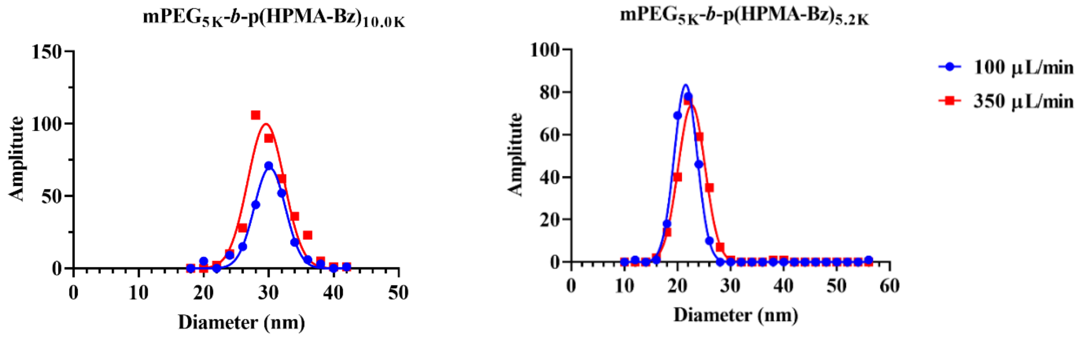

(**a**) (**b**)

Figure S14. Histograms of cryo-TEM diameters of (a) block copolymer copolymer B (mPEG$_{5K}$-*b*-p(HPMA-Bz)$_{10.0K}$)and (b) block copolymer C (mPEG$_{5K}$-*b*-p(HPMA-Bz)$_{5.2K}$) nanostructures prepared at 5 mg/ml polymer concentration and different flow rates. The data are fitted by Gaussian laws using GraphPad Prism.

Differential scanning calorimetry

Differential scanning calorimetry (DSC) was performed using a Discovery DSC (TA Instruments, New Castle, DE, USA) calibrated with indium. Samples (5-10 mg) were heated with a ramp of 2 °C/min up to 170 °C (modulated), kept isothermal for 2 min, cooled down at 1 ◦C/min to −90 °C (modulated), isothermal for 10 min, and subsequently heated at 2 °C /min up to 170 °C (modulated). The second heating cycle was used to obtain the glass transition temperature (T_g). T_g was analyzed by taking the point of inflection of the step change observed in the reversing heat flow curve. For all polymers the T_g is around 98 °C.

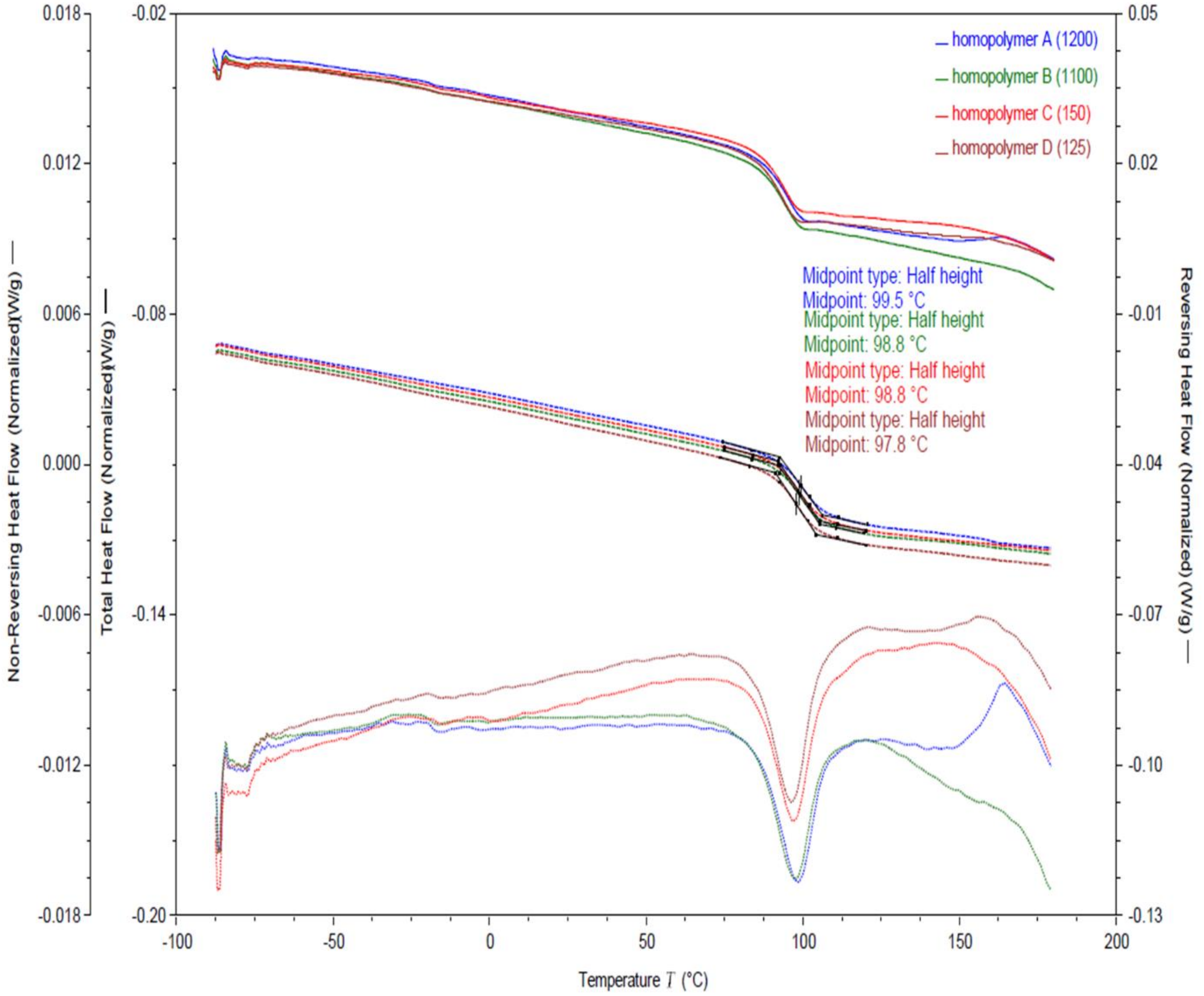

Figure S15. Thermograms of p(HPMA-Bz) homopolymers corresponding to the different molecular weight block copolymers recorded by DSC.

Author Contributions: Conceptualization W.E.H. and J.C.M.v.H.; investigation, J.B., M.B., C.L., S.L., I.A.B.P. and A.F.M.; writing—original draft preparation, J.B. and M.B.; writing—review and editing, J.B., M.B, C.L., O.S., S.L., S.A.M, C.F.v.N., W.E.H. and J.C.M.v.H.; supervision, S.L., O.S., S.A.M., C.F.v.N., W.E.H. and J.C.M.v.H.; funding acquisition, J.C.M.v.H.

All authors have read and agreed to the published version of the manuscript.

Funding: This research was funded by "the European Union's Horizon 2020 research and innovation program Marie Sklodowska-Curie Innovative Training Networks (ITN), grant number 676137 "NANOMED.

Acknowledgments: The CPER CAMPUSB project funded by the French state and the Region Nouvelle Aquitaine are gratefully acknowledged for acquisition of the Dolomite micromixer system. Mr Eric Laurichesse from Centre de Recherche Paul Pascal (CNRS, Univ. Bordeaux, France) and Dr Esra Aydinlioglu (LCPO) are kindly acknowledged for the helium pycnometry experiments to measure polymer mass density.

Conflicts of Interest: The authors declare no conflict of interest.

References

1. Micromixer Chip Available online: https://www.dolomite-microfluidics.com/product/micromixer-chip/ (accessed on May 26, 2020).

© 2020 by the authors. Submitted for possible open access publication under the terms and conditions of the Creative Commons Attribution (CC BY) license (http://creativecommons.org/licenses/by/4.0/).
